# Linking deep CO_2_ outgassing to cratonic destruction

**DOI:** 10.1093/nsr/nwac001

**Published:** 2022-01-08

**Authors:** Zhao-Xue Wang, Sheng-Ao Liu, Shuguang Li, Di Liu, Jingao Liu

**Affiliations:** State Key Laboratory of Geological Processes and Mineral Resources, China University of Geosciences, Beijing 100083, China; State Key Laboratory of Geological Processes and Mineral Resources, China University of Geosciences, Beijing 100083, China; State Key Laboratory of Geological Processes and Mineral Resources, China University of Geosciences, Beijing 100083, China; State Key Laboratory of Geological Processes and Mineral Resources, China University of Geosciences, Beijing 100083, China; State Key Laboratory of Geological Processes and Mineral Resources, China University of Geosciences, Beijing 100083, China

**Keywords:** carbonate metasomatism, lithospheric mantle, CO_2_ outgassing, deep carbon cycling, cratonic destruction, North China Craton

## Abstract

Outgassing of carbon dioxide from the Earth's interior regulates the surface climate through deep time. Here we examine the role of cratonic destruction in mantle CO_2_ outgassing via collating and presenting new data for Paleozoic kimberlites, Mesozoic basaltic rocks and their mantle xenoliths from the eastern North China Craton (NCC), which underwent extensive destruction in the early Cretaceous. High Ca/Al and low Ti/Eu and *δ*^26^Mg are widely observed in lamprophyres and mantle xenoliths, which demonstrates that the cratonic lithospheric mantle (CLM) was pervasively metasomatized by recycled carbonates. Raman analysis of bubble-bearing melt inclusions shows that redox melting of the C-rich CLM produced carbonated silicate melts with high CO_2_ content. The enormous quantities of CO_2_ in these magmas, together with substantial CO_2_ degassing from the carbonated melt–CLM reaction and crustal heating, indicate that destruction of the eastern NCC resulted in rapid and extensive mantle CO_2_ emission, which partly contributed to the early Cretaceous greenhouse climate episode.

## Teaser

Destruction of a craton undergoing pervasive carbonate metasomatism can release voluminous CO_2_ from the mantle, proposed by a combined study of Mg isotopes and melt inclusions on magmatism in the North China craton.

## INTRODUCTION

**Figure 1. fig1:**
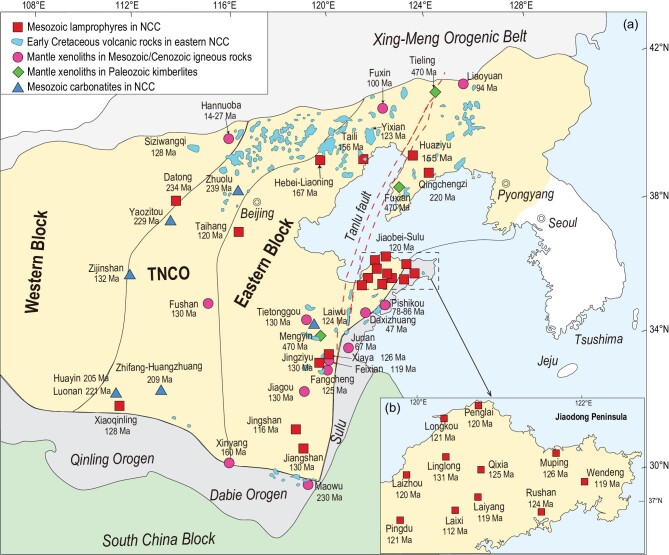
(a) Spatial distribution of Paleozoic diamond-bearing kimberlites, Mesozoic lamprophyres, carbonatites and mafic igneous rocks containing mantle xenoliths in the NCC (modified from ref. [36]). (b) The locations of early Cretaceous lamprophyres in the Jiaodong peninsula. More details and data sources are listed in the Supplementary Data.

Carbon exchange between the Earth's interior and exterior exerts an important influence on the surface climate through geologic time and is critical for planetary habitability. In recent years, it has been increasingly recognized that the cratonic lithospheric mantle (CLM) stores vast amounts of carbon, resulting from gradual enrichment by upward melt infiltration, in addition to the original carbon incorporated during its formation [1,2]. Carbon in the CLM can be extensively remobilized and released via continental rifting [1,3], active island arc volcanism [4,5] and plume-related magmatism [6,7], which represent three main ways proposed for mantle CO_2_ emission. For example, up to 28 to 34 Mt of carbon per year (expressed as Mt C yr^–1^) may be released by continental rifting [1]. A quantitative flux estimate for the CO_2_ outgassing along with the massive Tan-Lu Fault Belt in eastern China gave 70 ± 58 Mt C yr^–1^ [8].[Fig fig1] Extensive CO_2_ degassing (71 ± 33 Mt C yr^–1^) has been estimated through extensional faults along the entire East African Rift [3], which is even comparable to the estimates for CO_2_ degassing in island arcs (18–43 Mt C yr^–1^) and mid-ocean ridges (8–42 Mt C yr^–1^) [9–11]. Plume-induced CO_2_ outgassing has also been proposed to have the ability to have caused abrupt climate changes in Earth's history [12].

Cratons commonly retain tectonic and magmatic quiescence for billions of years [13], but some cratonic regions record extensive crustal deformation and on-craton magmatism that reflect cratonic destruction processes [14,15]. In sharp contrast to many others on Earth, the eastern part of the North China Craton (NCC) is a reactivated craton with the present-day lithosphere being made up of decoupled crust and mantle, i.e. Archean-Proterozoic crust and Phanerozoic lithospheric mantle [15]. The Archean thick (diamond-bearing) and cold lithospheric keel (>200 km) was partially or even wholly destroyed and removed, and was then replaced by a newly formed thin and hot lithospheric mantle (∼75 km), resulting in up to ∼120 km of the lithospheric keel being lost [14,16]. Reactivation of the CLM gave rise to a magmatic peak at ∼125 Ma including both mafic and felsic magmatism, marking the climax of cratonic lithospheric destruction in the early Cretaceous [16,17]. Since destruction/thinning is confined to the eastern part of the NCC (its western part remains largely intact), the westward subduction of the paleo-Pacific oceanic slab underneath the eastern Asian continent in the early Cretaceous was widely advocated to have resulted in reactivation and destruction of the eastern NCC [18].

In order to examine whether cratonic lithospheric destruction results in massive mantle CO_2_ outgassing, we firstly ascertain whether or not the CLM beneath the eastern NCC was initially carbon rich and whether it had been widely subjected to carbonate metasomatism, including carbon from recycled carbonates prior to destruction in the early Cretaceous. For this purpose,

we analyzed magnesium (Mg) isotopes for early Cretaceous lamprophyres and collated available chemical and Mg isotopic data for mantle xenoliths in Paleozoic diamond-bearing kimberlites and early Cretaceous mafic igneous rocks as well as orogenic ultramafic massifs in the Dabie orogen located on the south margin of the eastern NCC (Fig. 1). Then, we analyzed the CO_2_ components of melt inclusions (MIs) in early Cretaceous lamprophyres that can be used to calculate the CO_2_ concentrations in pre-eruptive magmas. Finally, we considered carbonated silicate melt–CLM reaction and crustal heating as additional ways for mantle CO_2_ outgassing to occur during cratonic destruction, in addition to mafic magmatism. Our results show that the CLM beneath the eastern NCC had widely interacted with carbonated melts prior to the early Cretaceous and extensive CO_2_ emission had occurred as a consequence of cratonic destruction.

## PRIMORDIAL CARBON IN THE CLM

The terrestrial mantle initially contained carbon resulting from accretion and core-mantle differentiation processes [11]. Recent studies provide a rigorous reconstruction of carbon concentration for the MORB source mantle and suggest that the upper mantle contains ∼30 ppm C [19]. In addition to the primordial carbon, the continental lithospheric mantle, mainly formed between 2 and 3 Ga, contains more carbon (∼89 ppm) that is incorporated into the sub-arc lithosphere via the accretion of island arcs [1]. Assuming an area of ∼1 000 000 km^2^ and lithospheric mantle thickness of ∼150 km for the eastern NCC prior to thinning in the Mesozoic, the CLM beneath the eastern NCC initially contains ∼4.27 × 10^7^ Mt C. In addition, the model of Foley and Fischer [1] predicts a long-term (>2 Ga) solid storage of carbon in the CLM as a result of episodic melt infiltration and redox freezing. If one considers this gradual enrichment from episodic freezing throughout the long evolution history of the ancient NCC (as old as ∼3.8 Ga; [20]), carbon concentration in the CLM beneath the eastern NCC may become much higher. Abundant diamonds were discovered in Paleozoic kimberlites from the NCC [21] and are direct evidence for a C-bearing, reduced CLM beneath the eastern NCC prior to the Mesozoic era. For example, the eruption of diamond-bearing kimberlites and the high Mg^#^ (>90; 100 × molar Mg/(Mg + Fe^2+^)) of olivines in diamond inclusions and xenolith/xenocryst olivines from Mengyin and Fuxian in the eastern NCC (Fig. 1) indicate the existence of a thick, low-density, cold root, which is mainly composed of refractory harzburgite and lherzolite [21].

## ADDITIONAL CARBON FROM RECYCLED CARBONATES

Since the Paleozoic, the NCC further underwent multiple oceanic plate subductions from the south, north and east sides, which potentially added surface carbon into the CLM beneath it. Below we present lines of evidence for more recent carbon addition to the CLM beneath the eastern NCC, from recycled carbonates related to slab subduction.

### Finding of low-***δ***^26^Mg lamprophyres

**Figure 2.**
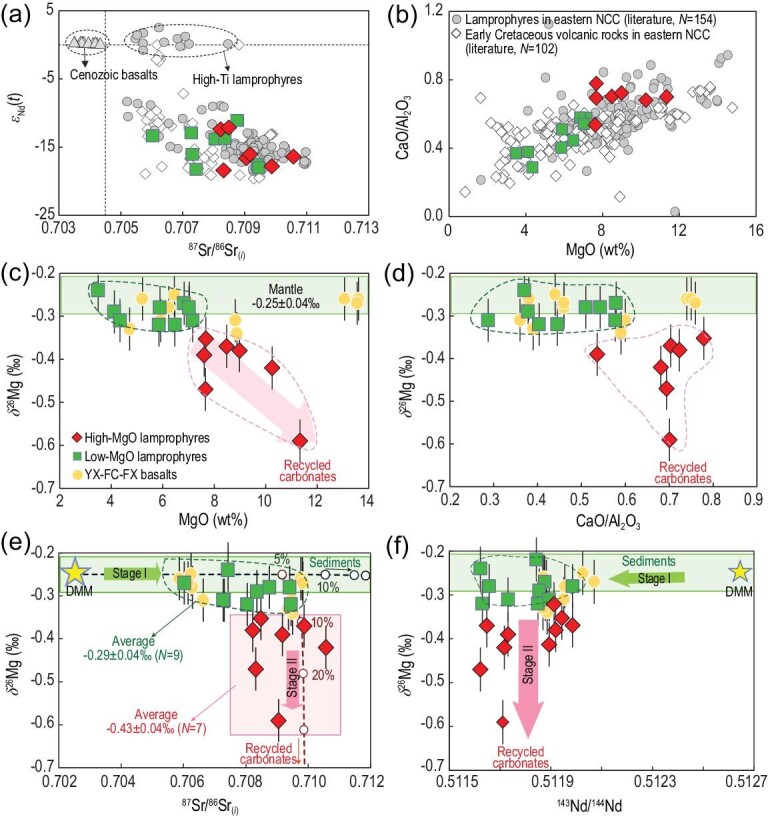
Plots of (a) ^87^Sr/^86^Sr*_(i)_* versus *ϵ*_Nd_*(t)* and (b) MgO versus CaO/Al_2_O_3_ for the lamprophyres, early Cretaceous volcanic rocks and Cenozoic basalts in the NCC. The gray circles represent the data of lamprophyres in previous studies, the gray triangles represent the data of Cenozoic basalts and the white diamonds represent the data of early Cretaceous volcanic rocks (all data are listed in Tables S1–S3). Plots of *δ*^26^Mg versus (c) MgO, (d) CaO/Al_2_O_3_, (e) ^87^Sr/^86^Sr*_(i)_* and (f) ^143^Nd/^144^Nd for the studied lamprophyres. Early Cretaceous basalts from Yixian, Feixian and Fangcheng (YX-FX-FC, yellow circles) in the eastern NCC [29] are shown for comparison. The magnesium isotopic composition of the terrestrial mantle (expressed as *δ*^26^Mg in per mil relative to DSM3) is from ref. [23]. The results of Mg-Sr isotopic modeling show a two-stage source metasomatism: the first stage is associated with siliciclastic sediments, and the second stage is related to carbonate metasomatism. The parameters used for Mg-Sr isotopic modeling are listed in the Supplementary Data (Table S4).

Magnesium isotopes are a novel and efficient tool for identifying recycled carbonates that are isotopically much lighter than the mantle [22,23]. Lamprophyres are typically characterized by a high content of volatiles and commonly record fluid/melt–mantle interaction in their magma sources [24], thereby providing an opportunity to investigate the nature of fluids/melts responsible for CLM metasomatism. Early Cretaceous lamprophyres are widely exposed in the NCC and have a magmatic peak at ∼125 Ma (Fig. 1), which is contemporaneous with the climax of cratonic lithospheric destruction of the eastern NCC [25]. Here we present a Mg isotopic dataset for Shandong lamprophyres, and for comparison we collate chemical and Sr-Nd isotopic data for other early Cretaceous lamprophyres widely distributed in the eastern NCC (Fig. 1; Tables S1–S3). Some High-Ti lamprophyres from Shandong have relatively depleted Sr and Nd isotopic compositions (Fig. 2a) and were proposed to have been derived from the asthenospheric mantle [26]. Most of the early Cretaceous lamprophyres in the eastern NCC, including those reported in this study, however, have extremely enriched Sr and Nd isotopic compositions (^87^Sr/^86^Sr*_(i)_* = 0.70520–0.71099, *ϵ*_Nd_*(t)* = −18.8 to −8.3) that are in sharp contrast to the High-Ti lamprophyres and Cenozoic alkali basalts in the NCC (Fig. 2a), pointing to an enriched CLM source. According to MgO contents, the Shandong lamprophyres are classified into Low-MgO (MgO < 7.5 wt%) and High-MgO (MgO > 7.5 wt%) subgroups (Fig. 2). Low-MgO lamprophyres have mantle-like *δ*^26^Mg (−0.32‰ to −0.24‰), whereas High-MgO lamprophyres possess significantly lower *δ*^26^Mg (−0.59‰ to −0.35‰) than the mantle *δ*^26^Mg value of −0.25 ± 0.04‰ (Fig. 2). It has been well demonstrated that Mg isotope fractionation during mantle partial melting and magma differentiation is limited (<0.07‰) [23]. In fact, the negative correlation between *δ*^26^Mg and MgO (Fig. 2c) argues against the light *δ*^26^Mg of the High-MgO lamprophyres being a result of fractional crystallization of any minerals involving removal of isotopically heavy Mg. More discussions about the influence of magma differentiation, as well as crustal contamination on Mg isotopic systematics of the studied lamprophyres, are provided in Supplementary Data. Overall, the variation of *δ*^26^Mg in Shandong lamprophyres reflects isotopically heterogeneous mantle sources caused by recycled crustal carbonates.

A carbonated mantle source for High-MgO lamprophyres is corroborated by their systematically higher CaO (High-MgO, 9.47 ± 0.84 wt%; Low-MgO, 7.25 ± 1.37 wt%; 1sd; Fig. S1) and CaO/Al_2_O_3_ ratios (High-MgO, 0.69 ± 0.07; Low-MgO, 0.45 ± 0.1; Fig. 2d) and lower Ni content (High-MgO, 81.96 ± 80.43 ppm; Low-MgO, 154.37 ± 81.89 ppm) in comparison with Low-MgO lamprophyres (Fig. S1), because marine carbonates are commonly Al and Ni poor and carbonate metasomatism can dramatically increase CaO of the mantle [27]. They also have distinct (Ti/Eu)_N_ ratios of 0.29 ± 0.03 (High-MgO; except for one sample with 0.53) and 0.43 ± 0.07 (Low-MgO), respectively. Mantle carbonate metasomatism also accounts well for the relatively low SiO_2_ of the High-MgO lamprophyres (Fig. S1) since partial melting of carbonated peridotites generates more Si-unsaturated melts relative to melting of volatile-poor peridotites [27]. The Mg-Sr isotopic mixing model (details listed in Table S4) suggests that the light-*δ*^26^Mg lamprophyres require source metasomatism by at least ∼10% recycled Mg-rich carbonates (e.g. dolomites; Fig. 2e). During slab subduction, Ca-rich carbonates can be substantially dissolved by aqueous fluids at initial stages and injected into the sub-arc mantle [9]. At larger depths of >160 km, dolomite dissolution occurs in subducting slabs and can be further enhanced by supercritical fluids [28]. The lithospheric mantle of the eastern NCC had a thickness of >200 km prior to thinning in the early Cretaceous [14,15]. Thus, the finding of low-*δ*^26^Mg lamprophyres demonstrates that the CLM beneath the eastern NCC had been metasomatized by dissolved magnesium carbonates from subducting slabs. The Low-MgO rocks are SiO_2_ rich and CaO poor and represent partial melts of the CLM metasomatized by recycled siliciclastic sediments, which explains their mantle-like *δ*^26^Mg yet highly radiogenic ^87^Sr/^86^Sr compositions (Fig. 2). We suggest a two-stage source metasomatism: the first stage is associated with siliciclastic sediments that led to the enriched Sr and Nd isotopic signatures, and the second stage is related to carbonate metasomatism that injected the low *δ*^26^Mg signatures, without significantly affecting Sr-Nd isotopic compositions (Fig. 2e and f). Previous studies found that early Cretaceous basalts from Fangcheng, Yixian and Feixian in the eastern NCC have mantle-like *δ*^26^Mg [29] (Fig. 2c and d). It is noteworthy that these basalts have CaO/Al_2_O_3_ and CaO/TiO_2_ ratios similar to those of the Low-MgO lamprophyres with normal *δ*^26^Mg. This indicates that the carbonated CLM may be chiefly sampled by lamprophyres that are commonly derived from a volatile-rich source [24]. Indeed, most of the Low-Ti lamprophyres with enriched Sr-Nd isotopic compositions from other regions in the eastern NCC have high CaO/Al_2_O_3_ and MgO content, which resembles the Shandong High-MgO lamprophyres with low *δ*^26^Mg (Fig. 2a and b). This implies that the pre-Cenozoic CLM beneath the eastern NCC may have undergone widespread metasomatism by recycled carbonates. Carbonate minerals (e.g. magnesite and calcite) are often observed in coeval lamprophyres in the NCC [30]. Overall, our new Mg isotopic data provide solid evidence for a recycled carbonate component in the CLM beneath the eastern NCC at or prior to the early Cretaceous.

### Evidence from xenoliths, ultramafic massifs and carbonatites

**Figure 3.**
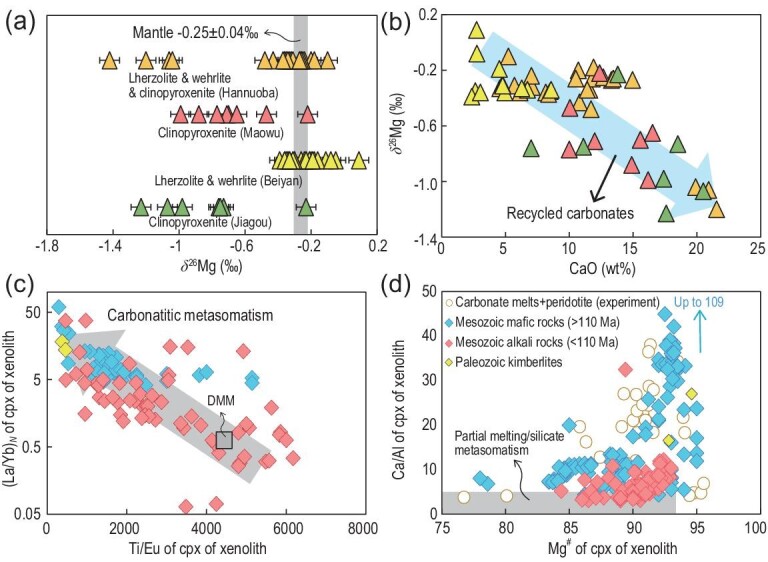
Compilation of available (a) Mg isotopic and (b, c, d) chemical data for mantle xenoliths hosted by Paleozoic diamond-bearing kimberlites and Mesozoic mafic igneous rocks in the NCC. Data for mantle xenoliths hosted by Cenozoic basalts (Hannuoba, Maowu, Beiyan) and Mesozoic intrusion (Jiagou) [28,34,35,57,58] are also shown (see text for details). Experimental data for carbonate melt–peridotite interaction are from ref. [36] and references therein. The data for depleted MORB source mantle (DMM) and clinopyroxene (CPX) of Paleozoic kimberlites, Mesozoic mafic rocks and Mesozoic alkaline rocks are summarized in Table S5.

Mantle-derived xenoliths in volcanic rocks and orogenic ultramafic massifs sample the lithospheric mantle, serving as a direct window to observe mantle metasomatism. A large number of lherzolite, wehrlite and clinopyroxenite xenoliths carried by Paleozoic diamond-bearing kimberlites, Mesozoic and Cenozoic mafic igneous rocks, and ultramafic massifs in the Dabie orogen derived from the deep mantle wedge (>160 km) on the south margin of the NCC (Fig. 1), record carbonate metasomatism of the CLM beneath the NCC. Wehrlites represent rocks where all, or most, orthopyroxene has been consumed through metasomatic reactions and are considered to be one of the end products of carbonate metasomatism in the CLM [8,31]. Pyroxenites and garnet pyroxenites represent rocks where all olivine and orthopyroxene have been consumed through metasomatic reactions with SiO_2_ carried by supercritical fluid or silica-rich melt and are therefore considered to be other end products of carbonate metasomatism in the CLM. Abundant wehrlite xenoliths have been found in Mesozoic basaltic rocks from Tietonggou and Liaoyuan (Fig. 1a) [32,33], which suggests pervasive carbonate metasomatism of the CLM. Pyroxenite xenoliths hosted by the Jiagou intrusion (∼130 Ma) in the southeastern NCC (Fig. 1) have extremely low *δ*^26^Mg of −1.23‰ to −0.73‰ (Fig. 3a) [34]. These pyroxenite xenoliths have a metasomatic U-Pb isotopic age of ∼400 Ma, suggesting carbonate metasomatism induced by paleo-Tethys slab subduction. Garnet pyroxenites in the Maowu ultramafic massif have low *δ*^26^Mg of −0.99‰ to −0.65‰ and contain abundant carbonate mineral inclusions and metasomatized zircons with high *δ*^18^O_SMOW_ (up to 12.2‰), suggesting metasomatism of the CLM by recycled carbonates. The age of zircons (457 ± 55 Ma) from the garnet clinopyroxenites also indicates Paleozoic metasomatism by subduction of the paleo-Tethys oceanic slab [28]. Some pyroxenite and garnet pyroxenite xenoliths hosted by Cenozoic basalts (e.g. Hannuoba) also have low *δ*^26^Mg [35] (Fig. 3a). Because Hannuoba is located to the west of the Daxing’anling-Taihang gravity lineament (DTGL) in the western part of the NCC, in which the CLM has not been affected by the Mesozoic thinning, the presence of low-*δ*^26^Mg xenoliths also indicates mantle carbonate metasomatism of the NCC prior to Cenozoic. It is noted that the Hannuoba xenoliths have *δ*^26^Mg (low to −1.42‰; Fig. 3a) much lower than those of the host basalts and all other Cenozoic basalts in eastern China (−0.6 to −0.3‰) [29], thus their low *δ*^26^Mg is unlikely to have been caused by interaction between low-*δ*^26^Mg basaltic melt and the overlying lithospheric mantle. Generally, there is a negative correlation between *δ*^26^Mg and CaO content for these xenoliths (Fig. 3b), strongly suggesting metasomatism of the CLM by recycled carbonates. Apart from Mg isotopes, Ca/Al, (La/Yb)_N_ and Ti/Eu ratios of clinopyroxenes are effective indices of mantle carbonatitic metasomatism. As shown in Fig. 3c and d, clinopyroxenes in mantle xenoliths hosted by Paleozoic diamond-bearing kimberlites and Mesozoic mafic igneous rocks have systematically higher Ca/Al and (La/Yb)_N_ and lower Ti/Eu ratios than those of silicate-metasomatic mantle xenoliths and depleted-MORB-mantle (DMM) peridotites. Along with high Mg^#^ and low Ti/Eu, these xenoliths are believed to have undergone carbonatitic metasomatism [36].

Further evidence for pre-Cenozoic carbonate metasomatism of the CLM comes from carbonatites. The solidus of mantle rocks can be reduced by addition of volatiles such as CO_2_ into the mantle and melting of the CO_2_-rich mantle would produce alkali-rich and silicon-poor melts, such as carbonatites [27]. Mesozoic carbonatites are exposed at more than 10 locations in the NCC [36–38] (Fig. 1). The enriched Sr and Nd isotopic compositions of these carbonatitic magmas suggest an enriched, carbonated mantle source [37]. Mesozoic carbonatites from Zhuolu and Huairen have high ^87^Sr/^86^Sr ratios (0.7055–0.7075) and are proposed to have formed by direct melting of recycled sedimentary carbonates in the mantle [38]. Carbonatites intruding on Neogene alkali basalts in Hannuoba on the northern margin of the NCC have high ^87^Sr/^86^Sr (0.70522–0.70796) and high *δ*^18^O ratios (22.2‰–23.0‰), which are directly linked to the subducted paleo-Asian oceanic slab beneath the NCC before the Mesozoic era [39].

**Figure 4.**
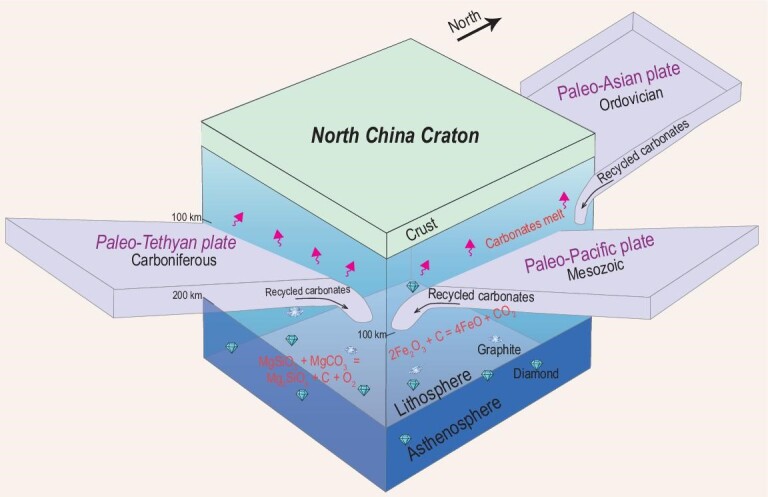
A cartoon representing multiple subduction events around the NCC at or prior to the early Cretaceous, including the paleo-Asian oceanic slab in the north since Ordovician, paleo-Pacific oceanic slab in the east since Mesozoic and paleo-Tethyan oceanic slab in the south beginning in the Carboniferous (modified from ref. [59]). Recycled carbonates would be reduced to diamond or graphite at depths of >150 km [40].

Collectively, the lines of evidence above strongly suggest that the CLM beneath the eastern NCC has been subject to pervasive carbonate metasomatism since the Paleozoic. The carbonate metasomatism could have been induced by multiple oceanic plate subduction events around the NCC, that is, the paleo-Asian oceanic slab in the north, paleo-Tethys oceanic slab in the south and paleo-Pacific slab in the east (Fig. 4). These subducted slabs carried large amounts of carbonate sediments into the mantle and transformed the CLM into a vast store for carbon. However, the deep part of the mantle is commonly too reduced to favor stable carbonates. That is, when carbonates are recycled into the mantle at depths of >120 km, they will be reduced via the following redox reaction:

$$\begin{equation*}
\begin{array}{@{}*{1}{c}@{}} \hspace*{-60pt}{{\rm{MgSi}}{{\rm{O}}_3} + {\rm{MgC}}{{\rm{O}}_3}\ = \ {\rm{M}}{{\rm{g}}_2}{\rm{Si}}{{\rm{O}}_4} + {\rm{C}} + {{\rm{O}}_2}.}\\
{{\rm{enstatite}}\quad {\rm{magnesite}}\quad {\rm{olivine}}\quad {\rm{diamond}}/{\rm{graphite}}} \end{array}
\end{equation*}$$

At depths of 120–170 km, recycled carbonate is transformed into carbon that exists as graphite and at larger depths (>170 km) as diamond [40], although in the CLM diamond is stable to lower pressures at cool conductive geotherms (Fig. 4). It is difficult to quantify the flux of recycled carbon in the mantle of the entire NCC since the Paleozoic, but we can give a rough estimate for this study area. As discussed above, the Mg-Sr isotopic mixing model indicates that the mantle source of low-*δ*^26^Mg lamprophyres contains ∼10 wt% Mg-rich carbonates (Fig. 2e), which is roughly equivalent to ∼1 wt% C. Assuming a density of 3.2 g cm^−3^ and a possible 40-km lithosphere depth interval that has been metasomatized, the mass of recycled C in the CLM beneath Shandong peninsula can be calculated. The lithosphere beneath the eastern NCC was >200 km thick before destruction [18,41] and the depth at which Mg-rich carbonates start to dissolve is ∼160 km [28,29]; thus we assume an ∼40-km interval for carbonate metasomatism. An areal estimate is available for the Shandong peninsula (∼73 000 km^2^), and we assume that about half the area was affected based on the proportion of occurrence of High-MgO lamprophyres with low *δ*^26^Mg in the study area (Fig. 2). From this, 6.09 × 10^7^ Mt C is estimated to have been added by recycled carbonates. Together with the primordial carbon (∼4.27 × 10^7^ Mt C) in the CLM prior to the Paleozoic, the total reservoir of carbon in the CLM beneath the eastern NCC would be 1.04 × 10^8^ Mt C at least, which represents a significant store of carbon in the CLM with important contribution from recycled carbonates. The reduced CLM, with carbon mainly existing as graphite or diamond, has not undergone redox melting and was preserved until the Mesozoic during which it was largely activated and removed.

## DEEP CO_2_ OUTGASSING INDUCED BY CRATONIC DESTRUCTION

**Figure 5. fig5:**
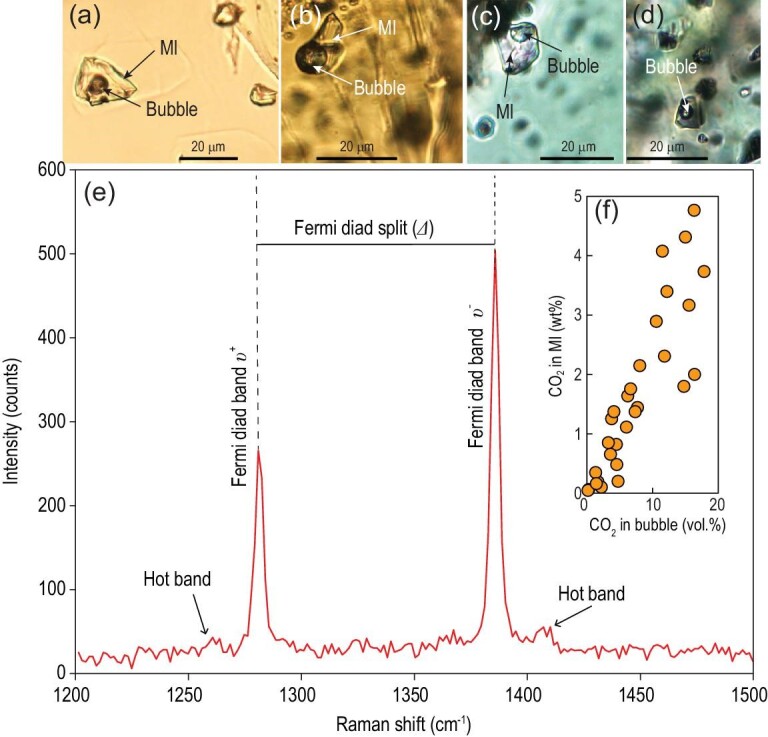
(a–d) Bubble-bearing MIs in the transmitted light optical microcopy. (e) Raman spectrum of a CO_2_-bearing bubble in a clinopyroxene-hosted MI from lamprophyres. The presence of CO_2_ is confirmed by the Fermi diad, consisting of two peaks at ∼1285 cm^–1^ and ∼1388 cm^–1^, bounded by hot bands, below 1285 cm^–1^ and above 1388 cm^–1^. (f) The relationship between the volume of CO_2_ in bubbles and the CO_2_ concentrations of MIs.

Commonly, the deep CLM is primarily reduced as a result of depletion in basaltic melt and the pressure effect on the oxygen fugacity during its formation [42]. A reduced CLM beneath the thick NCC (>200 km) is indicated by the Paleozoic diamond-bearing kimberlites. However, it can become oxidized as the diamondiferous CLM is exhumed to shallower depths due to lithospheric thinning, extension and mantle upwelling [8]. During this process, ‘redox melting’ would occur and carbon (diamond or graphite) in the CLM would become unstable and be oxidized by the reduction of Fe^3+^ at depths of <170 km [40]. This redox melting would produce carbonatitic melts at depths of ∼150 km that could evolve into carbonated silicate melts accompanied by silicate melting at shallower depths. The redox melting of the CLM was probably induced by the decompression and rise of the lithosphere–asthenosphere boundary due to slab rollback of the westward subducting paleo-Pacific plate at the early Cretaceous [18]. Carbonatitic melts have much lower viscosity and density relative to silicate melts [43], which could further promote carbonatite metasomatism of the shallower CLM. In the presence of carbonatitic melts, the mantle could be readily fusible, leading to efficient extraction of carbon from the deep interior [11]. Therefore, given the extensive thinning (>120 km) of the lithospheric mantle keel of the eastern NCC [14,16], extensive CO_2_ outgassing is expected to have occurred as the CLM underwent redox melting during this thinning process.[Fig fig5]

Here we evaluate whether or not the early Cretaceous lamprophyres are CO_2_ rich by analyzing gas exsolution bubble-bearing MIs in them. MIs are small droplets of silicate melt trapped by crystals in magmatic rocks and can be used to constrain the contents of volatile components dissolved in melts prior to volcanic eruption and, ideally, degassing. After the MIs are captured, bubbles will be formed during the cooling process of melts, post-entrapment crystallization on MI walls, or diffusive H^+^ loss [44]. Thus, the CO_2_ of MIs is present mainly in bubbles due to its low solubility in silicate melts if post-entrapment degassing occurs [44]. MIs are mainly hosted in clinopyroxene and occasionally in olivine and amphibole macrocrysts of the Shandong lamprophyres (Fig. S3). The compositions of gas exsolution bubbles were analyzed by Raman spectroscopy (see Supplementary Data for detailed methods). Among the ∼200 MIs we analyzed, >80% of the MIs contain vapor bubbles and ∼20% of the vapor bubbles contain CO_2_. The analyzed bubbles in most MIs are composed of pure or nearly pure CO_2_ without other volcanic gases (CO, CH_4_, H_2_S, H_2_O) being detected. The presence of CO_2_ in the bubbles of MIs was confirmed by two characteristic peaks, at ∼1285 cm^–1^ and ∼1388 cm^–1^, defining a Fermi diad in the Raman spectrum (Fig. 5). CO_2_ density (*d*) of the bubbles can be calculated by the spacing of the Fermi diad (*Δ* cm^–1^), using the equation of Kawakami *et al.* [45]. The mass of CO_2_ in bubbles can be calculated by multiplying CO_2_ density by the volume of the bubble (Table S6). Then, the CO_2_ content of the vapor bubble in ppm, [CO_2_]_vb_, can be calculated using the following equation [44]:

$$\begin{equation*}
{\left[ {{\rm{C}}{{\rm{O}}_2}} \right]_{{\rm{vb}}}} = {\rm{ }}\left( {{{\rm{M}}_{{\rm{vb}}}}^{{\rm{CO}}2}/{{\rm{M}}_{{\rm{gl}}}}} \right) \times {\rm{ }}{10^6},
\end{equation*}$$

where M_gl_ is the mass of glass within the MI, calculated as the glass volume multiplied by a melt density that is assumed to be 2.75 g cm^−3^ [46]. The results show that bubbles in MIs from the lamprophyres contain 323 to 47 490 ppm CO_2_ (*N *= 29), and >93% of bubbles have a CO_2_ content of >1000 ppm (0.1 wt%) (Fig. 5f; Table S6). The calculated CO_2_ concentrations in MIs of the lamprophyres range from 474 to 47 641 ppm (*N *= 29), with most (>80%) higher than 5000 ppm. Silicate crystal-hosted MIs, representing melts during various stages of an evolving magmatic system, can be analyzed to constrain the CO_2_ contents that dissolved in the melt before volcanic eruption and/or degassing [47]. We thus estimate that the measured CO_2_ concentrations represent those of the pre-eruptive and possibly evolved lamprophyre magmas, which mostly fall between 0.5 wt% and 2.0 wt%. These contents are similar to or even higher than the CO_2_ concentrations (0.5–1.0 wt%) in MIs of the end-Triassic Central Atlantic Magmatic Province basalts, which were estimated by the same method [48]. It should be noted that a high CO_2_ concentration of MIs is mainly observed in High-MgO lamprophyres with light *δ*^26^Mg values (see Supplementary Data), and the number of MIs in High-MgO lamprophyres is much larger than that in Low-MgO lamprophyres. This probably indicates that High-MgO lamprophyres with recycled carbonates in their mantle sources contain more MIs and higher CO_2_ concentrations in the pre-eruptive magmas, although low-volume melts could also have extremely high CO_2_ content even if the source is not specifically C rich, due to the strong incompatibility of CO_2_ in peridotite [49]. Because most of the early Cretaceous lamprophyres in the eastern NCC belong to the High-MgO group with low *δ*^26^Mg (Fig. 2), their sources were plausibly most strongly affected by carbonate metasomatism and attendant enrichment in carbon. Melting of this metasomatized CLM then produced primary magmas with high CO_2_ content, which may have been further enhanced during pre-eruptive differentiation. Here we collated geochemical data for early Cretaceous mantle-derived volcanic rocks (see Fig. 1 for locations, Fig. 2a and b and Fig. S4 for chemical compositions) and found that they are widely distributed in the eastern NCC and show similar geochemical characteristics to the lamprophyres. Thus, early Cretaceous mantle-derived magmas in the eastern NCC are much more abundant than those represented by lamprophyres. A larger flux of CO_2_ outgassing is thus expected during the period of extensive destruction of the NCC, in addition to lamprophyres. Intrusion or eruption of these magmas could have carried a large amount of CO_2_ from the mantle into the surface.

Experimental studies show that the solubility of carbon dioxide in melts decreases at lower pressures, and CO_2_ can even be directly degassed at mantle depths [50]. Therefore, the thinned lithosphere, as a result of cratonic destruction and extension, can further facilitate CO_2_ outgassing via magmatism. There is no evidence for the presence of a deep-sourced mantle plume beneath the eastern NCC during the Phanerozoic era. We thus propose that the destruction of the CLM represents another important cause of CO_2_ emission from the mantle, in addition to continental rifting, active island arc volcanism and mantle plume. During this process, carbon in the CLM can experience gradual oxidation during mantle upwelling, with a change of carbon speciation from a reduced to an oxidized form, and a portion of carbon can be liberated via redox melting from the reduced mantle [11].

Enormous CO_2_ reservoirs can be formed by the eruption or intrusion of magmas. There are abundant crustal CO_2_ reservoirs in the Songliao and Bohai Bay basins in the eastern NCC, which indicates that the volume of CO_2_ degassing is enormous [51]. CO_2_ reservoirs in the Songliao basin were formed primarily in Cretaceous, and voluminous inorganic CO_2_ (mainly mantle-derived and crust-derived) is observed in these reservoirs. For example, the high CO_2_ content (>90%) and *δ*^13^C (−4.95‰) and high helium isotopic composition (R/R_a _= 3.34) of Wanjinta reservoirs indicate that the CO_2_ was chiefly sourced from the mantle [51]. The Bohai Bay basin, a Mesozoic-Cenozoic basin, is the central area of destruction of the eastern NCC. The reservoirs there also have high CO_2_ content (79.17–98.61%) and high R/R_a_ (2–3.34), which indicates that the CO_2_ was derived from the mantle [51].

A recent study by Aulbach *et al.* [8] quantified the CO_2_ flux related to the reaction of the CLM with silica-undersaturated (carbonated) melt, referred to as wehrlitization, and linked this flux to surficial degassing in rifts and basins. As discussed above, abundant wehrlite and pyroxenite xenoliths are found in Mesozoic mantle-derived rocks in the eastern NCC, and many of these xenoliths have light Mg isotopic compositions and high Ca/Al ratios (Fig. 3). For instance, the characteristics of low Ti/Eu, high Ca/Al, (La/Yb)_N_ and Zr/Hf of clinopyroxenes in Liaoyuan wehrlites are ascribed to interaction with a silica-undersaturated, carbonated silicate melt [33]. These rocks thus record the substantial reaction between carbonated silicate melts and the CLM. In the CLM, these melts are initially out of thermal and compositional equilibrium, causing intensive melt-rock reactions. During this process, the following reaction will happen at ∼1.5–2.0 GPa [52]:

$$\begin{eqnarray*}
&& {\rm{Enstatite}} + {\rm{dolomite}}\left( {{\rm{melt}}} \right) = {\rm{forsterite}} \nonumber\\
&&\qquad +\, {\rm{diopside}} + {\rm{C}}{{\rm{O}}_2}\left( {{\rm{vapor}}}\right)\!.
\end{eqnarray*}$$

A quantitative estimate suggests that 2.9 to 10.2 kg CO_2_ can be released per 100 kg of wehrlite formed [8]. Extensive CO_2_ release is suggested to have occurred during the carbonated melt–CLM reaction process in the course of this destruction of the eastern NCC, along with the Tan-Lu Fault Belt, which was most active in the early Cretaceous [33].

**Figure 6.**
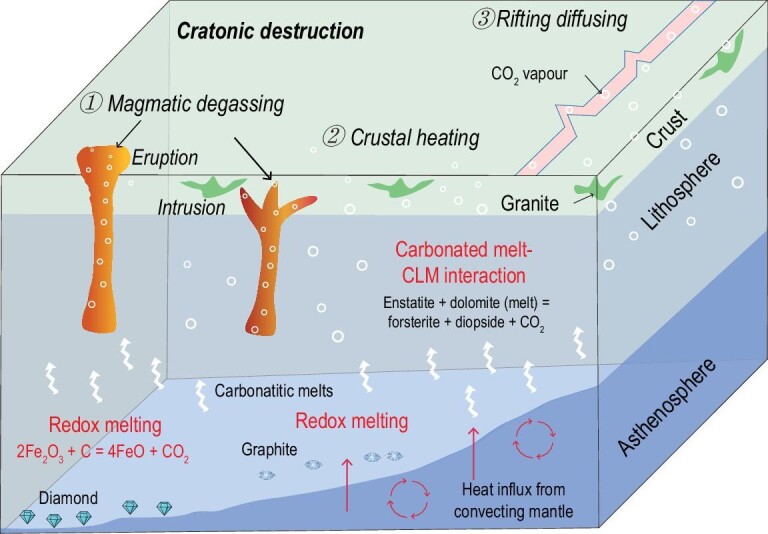
Schematic cartoon models illustrating mantle CO_2_ outgassing in response to cratonic destruction. The carbonated CLM beneath the eastern NCC underwent extensive melting and thinning at ∼125 Ma as a result of heat upwelling from mantle convection related to rollback of the subducting west Pacific slab. This process produced extensive CO_2_-rich magmas with a peak at ∼125 Ma, as recorded by lamprophyres, basalts and carbonatites. The CO_2_ vapors were released in three ways: ① magmatic degassing of lamprophyre and eruptive magmas, ② heating of sedimentary carbonates stored in the crust, represented by granites, and ③ the interaction between carbonated melts and the CLM, represented by wehrlites and pyroxenites leading to decarbonation and liberation of CO_2_ vapor.

Stable continents are long-term storage sites for sedimentary carbonates, and the amount of carbonates stored in continents is thought to be at least 10 times greater than that stored in oceanic crust [53]. Carbonates in crusts can be trapped by plutons that ascend to shallow levels in the arc crust or are transported into the lower crust during later arc stages. Global flare-ups in continental arc volcanism were proposed to have the potential to release CO_2_ as a result of magmatic interaction with ancient crustal carbonates stored in the continental crust [54,55]. The eastern NCC is typically characterized by a giant felsic magmatism event at the early Cretaceous with a volume much larger than that of mafic magmatism [17], implying large-scale crustal melting and reworking during the cratonic destruction process. These early Cretaceous felsic magmas (i.e. granites) have high zirconium saturation temperatures and contain an important contribution from the hot upwelling mantle [25]. Decarbonation is expected to widely occur during interaction between the hot felsic magmas and the limestones chronically stored in the continental crust. This process could also contribute to CO_2_ release, in addition to mantle CO_2_ outgassing via mafic magmatism and carbonated melt–CLM reaction during destruction of the eastern NCC (Fig. 6).

## POSSIBLE CONTRIBUTIONS TO THE CRETACEOUS GREENHOUSE

The amount of CO_2_ outgassing induced by the destruction of the eastern NCC could be significant, particularly if one considers that the removed CLM contained a large amount of recycled carbon from subducted slabs prior to thinning. The fluxes of deep CO_2_ outgassing during this destruction process may be large given the short duration of mantle-derived, CO_2_-rich magmatism (both intrusions and volcanics; Fig. 1) in the early Cretaceous (∼125 Ma) as well as the strong carbonated silicate melt–CLM reaction that resulted in substantial CO_2_ release along the massive Tan-Lu Fault Belt [8]. At a larger scale, enormous quantities of CO_2_ that were rapidly released into the atmosphere, induced by the destruction, may have perturbed the global climate and partly contributed to the atmospheric CO_2_ rise during the Cretaceous, one of the longest greenhouse periods of Earth's history, with atmospheric CO_2_ levels 4 to 10 times higher than those prior to the Industrial Revolution [56].

## CONCLUSIONS

We present the first Mg isotope data for early Cretaceous lamprophyres and collate available chemical and Mg isotopic data for mantle xenoliths in Paleozoic diamond-bearing kimberlites and Mesozoic mafic igneous rocks as well as orogenic ultramafic massifs in the NCC. These results suggest the presence of a widespread, C-rich CLM beneath the NCC at and before lithospheric thinning in the early Cretaceous, with an important contribution from recycled carbonate sediments. Long-term and three-sided—i.e. the south, north and east—oceanic plate subductions underneath the NCC during the Paleozoic and Mesozoic could have contributed a vast amount of carbon to the lithospheric mantle of the NCC. Redox melting of the reduced, C-rich CLM as it was exhumed to shallower depths due to lithospheric extension and thinning in the early Cretaceous generated large amounts of basaltic lavas and lamprophyres, resulting in the release of voluminous CO_2_ to the exosphere. This may represent an important cause of CO_2_ emission from the mantle, in addition to mantle plumes, active island arc volcanism and continental rifts, as proposed in previous studies [1,3–7]. The amount of magmatic CO_2_ outgassing is largely supplemented by the release of mantle CO_2_ induced by the carbonated melt–CLM reaction [8] and decarbonation induced by interaction between hot felsic magmas and crustal limestones. Therefore, deep CO_2_ outgassing can be linked to the destruction of a long-term stable craton and can be said to have enhanced global CO_2_ input into the atmosphere.

## Supplementary Material

nwac001_Supplemental_File

## References

[1] Foley SF , Fischer TP. An essential role for continental rifts and lithosphere in the deep carbon cycle. Nat Geosci 2017; 10: 897–902. 10.1038/s41561-017-0002-7

[2] Plank T , Manning CE. Subducting carbon. Nature 2019; 574: 343–52. 10.1038/s41586-019-1643-z 31619791

[3] Lee H , Muirhead JD, Fischer TP et al. Massive and prolonged deep carbon emissions associated with continental rifting. Nat Geosci 2016; 9: 145–9. 10.1038/ngeo2622

[4] Burton MR , Sawyer GM, Granieri D. Deep carbon emissions from volcanoes. Rev Mineral Geochem 2013; 75: 323–54. 10.2138/rmg.2013.75.11

[5] Marty B , Tolstikhin IN. CO_2_ fluxes from mid-ocean ridges, arcs and plumes. Chem Geol 1998; 145: 233–48. 10.1016/S0009-2541(97)00145-9

[6] Barnet JSK , Littler K, Kroon D et al. A new high-resolution chronology for the late Maastrichtian warming event: establishing robust temporal links with the onset of Deccan volcanism. Geology 2018; 46: 147–50. 10.1130/G39771.1

[7] Schoene B , Eddy MP, Samperton KM et al. U-Pb constraints on pulsed eruption of the Deccan Traps across the end-Cretaceous mass extinction. Science 2019; 363: 862–6. 10.1126/science.aau2422 30792300

[8] Aulbach S , Lin AB, Weiss Y et al. Wehrlites from continental mantle monitor the passage and degassing of carbonated melts. Geochem Perspect Lett 2020; 15: 30–4. 10.7185/geochemlet.2031

[9] Kelemen PB , Manning CE. Reevaluating carbon fluxes in subduction zones, what goes down, mostly comes up. Proc Natl Acad Sci USA 2015; 112: E3997–4006. 10.1073/pnas.1507889112 26048906 PMC4522802

[10] Kagoshima T , Sano Y, Takahata N et al. Sulphur geodynamic cycle. Sci Rep 2015; 5: 8330. 10.1038/srep08330 25660256 PMC4321164

[11] Dasgupta R , Hirschmann MM. The deep carbon cycle and melting in Earth's interior. Earth Planet Sci Lett 2010; 298: 1–13. 10.1016/j.epsl.2010.06.039

[12] Sobolev SV , Sobolev AV, Kuzmin DV et al. Linking mantle plumes, large igneous provinces and environmental catastrophes. Nature 2011; 477: 312–6. 10.1038/nature10385 21921914

[13] Pearson DG , Scott JM, Liu J et al. Deep continental roots and cratons. Nature 2021; 596: 199–210. 10.1038/s41586-021-03600-5 34381239

[14] Griffin WL , Zhang A, O’Reilly SY et al. Phanerozoic evolution of the lithosphere beneath the Sino-Korean Craton. In: Flower M, Chung S, Luo C et al. (eds.). Mantle Dynamics and Plate Interactions in East Asia. Washington DC: American Geophysical Union, 1998, 107–26. Doi: 10.1130/0091-7613(1992)020≥0339:ROMCIT≤2.3.CO;2

[15] Menzies MA , Fan W, Zhang M. Palaeozoic and Cenozoic lithoprobes and the loss of >120 km of Archaean lithosphere, Sino-Korean craton, China. Geol Soc Spec Publ 1993; 76: 71–81. 10.1144/GSL.SP.1993.076.01.04

[16] Menzies M , Xu Y, Zhang H et al. Integration of geology, geophysics and geochemistry: a key to understanding the North China Craton. Lithos 2007; 96: 1–21. 10.1016/j.lithos.2006.09.008

[17] Wu F-Y , Yang J-H, Wilde SA et al. Geochronology, petrogenesis and tectonic implications of Jurassic granites in the Liaodong Peninsula, NE China. Chem Geol 2005; 221: 127–56. 10.1016/j.chemgeo.2005.04.010

[18] Zhu R , Xu Y, Zhu G et al. Destruction of the North China Craton. Sci China Earth Sci 2012; 55: 1565–87. 10.1007/s11430-012-4516-y

[19] Marty B. The origins and concentrations of water, carbon, nitrogen and noble gases on Earth. Earth Planet Sci Lett 2012; 313–14: 56–66. 10.1016/j.epsl.2011.10.040

[20] Liu DY , Nutman AP, Compston W et al. Remnants of ≥ 3800 Ma crust in the Chinese part of the Sino-Korean craton. Geology 1992; 20: 339–42. 10.1130/0091-7613(1992)020≥0339:ROMCIT≤2.3.CO;2

[21] Zheng J. Comparison of mantle-derived materials from different spatiotemporal settings: implications for destructive and accretional processes of the North China Craton. Sci Bull 2009; 54: 3397–416. 10.1007/s11434-009-0308-y

[22] Liu S-A , Li S-G. Tracing the deep carbon cycle using metal stable isotopes: opportunities and challenges. Engineering 2019; 5: 448–57. 10.1016/j.eng.2019.03.007

[23] Teng F-Z. Magnesium isotope geochemistry. Rev Mineral Geochem 2017; 82: 219–87. 10.2138/rmg.2017.82.7

[24] Rock NMS. The nature and origin of lamprophyres: an overview. Geol Soc Spec Publ 1987; 30: 191–226. 10.1144/GSL.SP.1987.030.01.09

[25] Wu F-Y , Yang J-H, Xu Y-G et al. Destruction of the North China Craton in the Mesozoic. Annu Rev Earth Planet Sci 2019; 47: 173–95. 10.1146/annurev-earth-053018-060342

[26] Ma L , Jiang S-Y, Hofmann AW et al. Lithospheric and asthenospheric sources of lamprophyres in the Jiaodong Peninsula : a consequence of rapid lithospheric thinning beneath the North China Craton? Geochim Cosmochim Acta 2014; 124: 250–71. 10.1016/j.gca.2013.09.035

[27] Dasgupta R , Hirschmann MM, Smith ND. Partial melting experiments of peridotite + CO_2_ at 3 GPa and genesis of Alkalic ocean island basalts. J Petrol 2007; 48:2093–124. 10.1093/petrology/egm053

[28] Shen J , Li S-G, Wang S-J et al. Subducted Mg-rich carbonates into the deep mantle wedge. Earth Planet Sci Lett 2018; 503: 118–30. 10.1016/j.epsl.2018.09.011

[29] Li S-G , Yang W, Ke S et al. Deep carbon cycles constrained by a large-scale mantle Mg isotope anomaly in eastern China. Natl Sci Rev 2017; 4: 111–20. 10.1093/nsr/nww070

[30] Ma L , Jiang S-Y, Hofmann AW et al. Rapid lithospheric thinning of the North China Craton: new evidence from cretaceous mafic dikes in the Jiaodong Peninsula. Chem Geol 2016; 432: 1–15. 10.1016/j.chemgeo.2016.03.027

[31] Neumann ER , Wulff-Pedersen E, Pearson NJ et al. Mantle xenoliths from Tenerife (Canary Islands): evidence for reactions between mantle peridotites and silicic carbonatite melts inducing Ca metasomatism. J Petrol 2002; 43:825–57. 10.1093/petrology/43.5.825

[32] Zhou Q , Xu W, Yang D et al. Modification of the lithospheric mantle by melt derived from recycled continental crust evidenced by wehrlite xenoliths in Early Cretaceous high-Mg diorites from western Shandong, China. Sci China Earth Sci 2012; 55: 1972–86. 10.1007/s11430-012-4533-x

[33] Lin AB , Zheng JP, Aulbach S et al. Causes and consequences of wehrlitization beneath a trans-lithospheric fault: evidence from mesozoic basalt-borne wehrlite xenoliths from the Tan-Lu fault belt, North China Craton. J Geophys Res Solid Earth 2020; 125: e2019JB019084. 10.1029/2019JB019084

[34] Wang Z-Z , Liu S-A, Ke S et al. Magnesium isotopic heterogeneity across the cratonic lithosphere in eastern China and its origins. Earth Planet Sci Lett 2016; 451: 77–88. 10.1016/j.epsl.2016.07.021

[35] Hu Y , Teng F-Z, Zhang H-F et al. Metasomatism-induced mantle magnesium isotopic heterogeneity: evidence from pyroxenites. Geochim Cosmochim Acta 2016; 185: 88–111. 10.1016/j.gca.2015.11.001

[36] Zong K , Liu Y. Carbonate metasomatism in the lithospheric mantle: implications for cratonic destruction in North China. Sci China Earth Sci 2018; 61: 711–29. 10.1007/s11430-017-9185-2

[37] Ying J. Geochemical and isotopic investigation of the Laiwu-Zibo carbonatites from western Shandong Province, China, and implications for their petrogenesis and enriched mantle source. Lithos 2004; 75: 413–26. 10.1016/j.lithos.2004.04.037

[38] Yan G , Mu B, Zeng Y et al. Igneous carbonatites in North China craton: the temporal and spatial distribution, Sr and Nd isotopic characteristics and their geological significance (in Chinese). Geol J China Univ 2007; 13: 463–73.

[39] Chen C , Liu Y, Foley SF et al. Paleo-Asian oceanic slab under the North China craton revealed by carbonatites derived from subducted limestones. Geology 2016; 44: 1039–42. 10.1130/G38365.1

[40] Stagno V , Ojwang DO, McCammon CA et al. The oxidation state of the mantle and the extraction of carbon from Earth's interior. Nature 2013; 493: 84–8. 10.1038/nature11679 23282365

[41] Xu Y. Thermo-tectonic destruction of the Archaean lithospheric keel beneath the Sino-Korean craton in China: evidence, timing and mechanism. Phys Chem Earth Part A 2001; 26: 747–57. 10.1016/S1464-1895(01)00124-7

[42] Frost DJ , McCammon CA. The redox state of Earth's mantle. Annu Rev Earth Planet Sci 2008; 36: 389–420. 10.1146/annurev.earth.36.031207.124322

[43] Kono Y , Kenney-Benson C, Hummer D et al. Ultralow viscosity of carbonate melts at high pressures. Nat Commun 2014; 5: 5091. 10.1038/ncomms6091 25311627

[44] Hartley ME , Maclennan J, Edmonds M et al. Reconstructing the deep CO_2_ degassing behaviour of large basaltic fissure eruptions. Earth Planet Sci Lett 2014; 393: 120–31. 10.1016/j.epsl.2014.02.031

[45] Kawakami Y , Yamamoto J, Kagi H. Micro-Raman densimeter for CO_2_ inclusions in mantle-derived minerals. Appl Spectrosc 2003; 57: 1333–9. 10.1366/000370203322554473 14658145

[46] Passmore E , Maclennan J, Fitton G et al. Mush disaggregation in basaltic magma chambers: evidence from the ad 1783 Laki eruption. J Petrol 2012; 53: 2593–623. 10.1093/petrology/egs061

[47] Steele-MacInnis M , Esposito R, Moore LR et al. Heterogeneously entrapped, vapor-rich melt inclusions record pre-eruptive magmatic volatile contents. Contrib Mineral Petrol 2017; 172: 18. 10.1007/s00410-017-1343-3

[48] Capriolo M , Marzoli A, Aradi LE et al. Deep CO_2_ in the end-Triassic Central Atlantic Magmatic Province. Nat Commun 2020; 11: 1670. 10.1038/s41467-020-15325-6 32265448 PMC7138847

[49] Hirschmann MM . Partial melt in the oceanic low velocity zone. Phys Earth Planet Inter 2010; 179: 60–71. 10.1016/j.pepi.2009.12.003

[50] Boudoire G , Rizzo AL, Di Muro A et al. Extensive CO_2_ degassing in the upper mantle beneath oceanic basaltic volcanoes: first insights from Piton de la Fournaise volcano (La Réunion Island). Geochim Cosmochim Acta 2018; 235: 376–401. 10.1016/j.gca.2018.06.004

[51] Zhao F , Jiang S, Li S et al. Correlation of inorganic CO_2_ reservoirs in East China to subduction of (Paleo-)Pacific Plate (in Chinese). Earth Sci Front 2017; 24: 370–84.

[52] Yaxley GM , Green DH, Kamenetsky V. Carbonatite metasomatism in the Southeastern Australian lithosphere. J Petrol 1998; 39: 1917–30. 10.1093/petroj/39.11-12.1917

[53] Lee CTA , Lackey JS. Global continental arc flare-ups and their relation to long-term greenhouse conditions. Elements 2015; 11: 125–30. 10.2113/gselements.11.2.125

[54] McKenize NR , Horton BK, Loomis SE et al. Continental arc volcanism as the principal driver of icehouse-greenhouse variability. Science 2016; 352: 444–7. 10.1126/science.aad5787 27102480

[55] Mason E , Edmonds M, Turchyn AV. Remobilization of crustal carbon may dominate volcanic arc emissions. Science 2017; 357: 290–4. 10.1126/science.aan5049 28729507

[56] Huber BT , Norris RD, MacLeod KG. Deep-sea paleotemperature record of extreme warmth during the Cretaceous. Geology 2002; 30: 123–6. 10.1130/0091-7613(2002)030≥0123:DSPROE≤2.0.CO;2

[57] Xiao Y , Teng F-Z, Zhang H-F et al. Large magnesium isotope fractionation in peridotite xenoliths from eastern North China craton: product of melt–rock interaction. Geochim Cosmochim Acta 2013; 115: 241–61. 10.1016/j.gca.2013.04.011

[58] Hu J , Jiang N, Carlson RW et al. Metasomatism of the crust-mantle boundary by melts derived from subducted sedimentary carbonates and silicates. Geochim Cosmochim Acta 2019; 260: 311–28. 10.1016/j.gca.2019.06.033

[59] Xiao Y , Teng F-Z, Su B-X et al. Iron and magnesium isotopic constraints on the origin of chemical heterogeneity in podiform chromitite from the Luobusa ophiolite, Tibet. Geochem Geophys Geosyst 2016; 17: 940–53. 10.1002/2015GC006223

