# Supplementary material for: Linking deep CO2 outgassing to cratonic destruction
Source: Natl Sci Rev. 2022 Jan 8;9(6):nwac001. doi: 10.1093/nsr/nwac001 (PMC9166544; doi:10.1093/nsr/nwac001)
Supplement: nwac001_Supplemental_File [file nwac001_supplemental_file.pdf]

**National Science Review**

Supplementary Data for

# Linking deep $CO_2$ outgassing to cratonic destruction

Zhao-Xue Wang, Sheng-Ao Liu*, Shuguang Li*, Di Liu, Jingao Liu

State Key Laboratory of Geological Processes and Mineral Resources, China University of Geosciences, Beijing 100083, China

*Corresponding to Sheng-Ao Liu (lsa@cugb.edu.cn) or Shuguang Li (lsg@ustc.edu.cn)

**Contents of this file:**

Geological settings and sample description

Analytical methods

Discussion on the effects of crustal contamination and magma differentiation

Methods for calculating $CO_2$ contents in MIs

Figures S1, S2, S3, S4

Tables S1, S2, S3, S4, S5, S6

## GEOLOGICAL SETTINGS AND SAMPLE DESCRIPTION

The Sino-Korean or North China craton, one of the world's oldest cratons, is composed of a Neoarchean to Paleoproterozoic basement with ages of crustal rocks up to 3.8 Ga [1]. The NCC consists of three parts: the Eastern Block, the Western Block, and the Trans-North China Orogen (TNCO) or called Central Orogenic belt in the middle (Fig. 1). The TNCO is a broadly north-south trending belt, which was formed at ca. 1.85 Ga through the collision of the Eastern and Western Blocks [2,3]. This collision event represents the final cratonization of the NCC. Since the Ordovician or later, the Eastern Block of the NCC has been reactivated and thinned by various thermal, chemical and/or tectonic processes [4], and the thick, cold and refractory lithospheric mantle beneath the eastern NCC in the Paleozoic was replaced by a thin, hot and fertile continental lithospheric mantle (CLM) in the Cenozoic [5-9]. The destruction/thinning of the cratonic lithospheric mantle (CLM) beneath the eastern NCC was accompanied with generation of extensive mafic and felsic magmatism in the Mesozoic and Cenozoic, e.g., in the Shandong Peninsula and other areas. Mafic dykes (e.g., lamprophyres and dolerite-porphyries) intruded into Mesozoic granitoids and Archaean metamorphic basement rocks in the Shandong Peninsula, ranging in age from 131 Ma to 112 Ma with a magmatic peak of ca. 125 Ma (Fig. 1).

A total of 16 lamprophyre samples were sampled from five locations (Pingdu, Laizhou, Linglong, Qixia and Haiyang) that cover the majority of lamprophyre outcrops in the Shandong Peninsula. Because of the presence of an obvious contact boundary between lamprophyres and wall rocks, the samples were collected at the core of the dikes so as to minimize the effect of interaction with wall rocks. Most of these collected rocks are unaltered, melanic and hypabyssal rocks, composed of hypidiomorphic to fine grains with porphyritic texture or porphyaceous-porphyritic texture. Phenocrysts consist mainly of amphibole (~5%), clinopyroxene (~10%) and plagioclase (~5%), and the groundmass includes amphibole (~15%), plagioclase (~40%), clinopyroxene (~15%) and biotite (~10%).

# ANALYTICAL METHODS

## Major and trace elements

Major element analysis of whole rocks was performed by using Inductively Coupled Plasma-Atomic Emission Spectrometry (ICP-AES) at the China University of Geosciences, Beijing (CUGB). The procedure was carried out by heating the sample powder at 980 $^{o}$C over half an hour to obtain the loss on ignition (LOI) firstly, followed by mixing about 0.4 g of samples with Li-tetraborate ($Li_2B_4O_7$) and fusing the samples in a Pt-(Au) crucible at about 1000 $^{o}$C. The analytical uncertainties are better than ±2% (2σ). Trace element contents were measured using inductively coupled plasma mass spectrometer (ICP-MS) at the CUGB. Approximately 50 mg of sample powder was dissolved in a Teflon bomb at 190 $^{o}$C by adding the mixed acids of concentrated ~14 N $HNO_3$ + ~29 N HF. 1 ml concentrated $HNO_3$ was added to the sample after the dissolved solution was dried at 140 $^{o}$C. The residue was re-dissolved in 50 ml 3% $HNO_3$ that contains 5 ppb indium. The analysis reproducibility of elements with high concentrations (>10 μg/g) was better than 10% (2σ) and that for elements with low concentrations (<10 μg/g) was better than ~20% (2σ). The data of reference materials, which were measured along with studied samples, are similar with recommended values (Table S3).

## Sr-Nd-Mg isotopes

Sample dissolution and chemical purification of Sr and Nd were performed using ultrapure water (>18.2 MΩ) and other distilled reagents in lamellar flow hoods (Class 100) inside the ultra-clean room (Class 1000), accompanied with percolated air at CUGB. About 50–70 mg of whole-rock powder was dissolved in FTFE beakers (Savillex®) by adding a mixture of $HNO_3$, HCl, and HF acids. The solution was evaporated to dryness and 1 ml 2.5 N HCl was added in order to prepare for ion exchange chromatography. Finally, the homogeneous solution was loaded on a vitreous column filled with 2 ml Bio-Rad cation resin AG50W-X12 that was pre-cleaned to separate Sr and Nd from other matrices (e.g., Mg, Fe, Sm), and Nd

was collected by LN resin, following the methods of Xu et al. [10]. The whole procedural blank is less than 0.1 and 0.05 ng for Sr and Nd, respectively. The Sr and Nd isotope compositions were measured on a Thermo Scientific *Neptune plus* multi-collector inductively coupled plasma mass spectrometer (MC-ICP-MS). The long-term analysis of the reference material BHVO-2 in our lab yielded $^{87}Sr/^{86}Sr$ = 0.703503 ±13 (2σ) and $^{143}Nd/^{144}Nd$ = 0.512979 ±9 (2σ). The data of reference materials (BHVO-2, BCR-2) that were measured in this study are listed in Table S1.

The procedure for Mg purification from matrices included processing in laminar flow hoods. About 5 mg homogenized sample powder was digested in FTFE beakers (Savillex®) with 1.5 ml HF and 0.5 ml $HNO_3$, aqua regia, and 1 ml concentrated $HNO_3$, sequentially. After complete dissolution, the sample solution was desiccated to dryness at 130 °C and finally dissolved in 0.1 ml 1 N $HNO_3$ to prepare for chemical separation. Purification of Mg from matrix elements was conducted by cation chromatography using AG50W-X8 (Bio-Rad 200 – 400 mesh) pre-cleaned resin, and Mg was eluted in the last 15 ml 1 N $HNO_3$. Detailed analytical procedures were reported in [11] and reference therein. Briefly, 100 μl solution containing about 20 μg of Mg was loaded into the column filled up with resin and eluted with 1 N $HNO_3$, and the same column procedure mentioned above was repeated twice. The whole procedural blank is less than 10 ng. Magnesium isotope ratios were analyzed by a Thermo Scientific *Neptune plus* MC-ICP-MS in a low-resolution mode. The method of standard-sample bracketing (SSB) was used to correct the instrumental mass bias. Each measurement was conducted for one block of 40 cycles, and each sample was measured 3 or 4 times. Two rock reference (BHVO-2 and BCR-2) standards and GSB Mg (an ultrapure Mg solution from the China Iron and Steel Research Institute) were analyzed along with samples to assess the precision and accuracy of this process. The Mg isotope compositions of samples are reported in the $\delta$-notation against the Mg standard DSM-3 [12]. The long-term external reproducibility in this laboratory for $\delta^{26}Mg$ is ± 0.06‰ (2SD). The $\delta^{26}Mg$ of BHVO-2 and BCR-2 (international basalt standards)

measured in this study are -0.24 ± 0.04‰ (2SD) and -0.12 ± 0.04‰ (2SD), respectively, which are consistent with the recommended values in the literature [11,13].

### Raman microspectroscopy analysis

Raman spectroscopy is a versatile non-destructive technique for melt/fluid inclusion ananlysis. $CO_2$ bubbles in melt inclusions were detected by Laser Raman spectrometer at CUGB. Analyses were performed by a laser with wavelength 514 nm, and an OLYMPUS ×100 objective was used to focus the laser on the analysed sites to obtain Raman spectra of melt inclusions at ambient temperature conditions. The investigated spectra range from 100 to 4000 $cm^{-1}$, depending on the spectral region of interest for each analysed phase. To minimise the signal-to-noise ratio, each ananlysis was comprised by 20 s counting time. The transverse resolution of measurements is less than 0.5 μm and the longitudinal resolution is less than 1 μm. The reproducibility of spectral curve is ± 0.2 $cm^{-1}$. All Raman data were processed through Origin software.

Clinopyroxene macrocrysts with sizes ranging from hundreds of μm to several mm were picked out from crushed lamprophyre samples including High-MgO (sample No. HY04, HY11 and QX01) and Low-MgO (QX03) lamprophyres. Unaltered clinopyroxenes containing glassy melt inclusions without post-entrapment daughter crystals were chosen to mount in CrystalBond and polished to expose flat crystal surface without breaching the melt inclusions. Single or multi melt inclusions (MIs) can be found in nearly all clinopyroxenes, and the MIs usually have irregular shapes and contain a single bubble. The sizes of melt inclusions and $CO_2$-bearing bubbles were measured by high resolution and high magnification photomicrographs. The lengths and widths of these MIs range from several μm to dozens of μm.

## CRUSTAL CONTAMINATION AND MAGMA DIFFERENTIATION

Shandong lamprophyres in this study are characterized by high $^{87}Sr/^{86}Sr_{(i)}$ and low $\varepsilon_{Nd}(t)$ values (Table S3). Similar geochemical characteristics have also been observed in early Cretaceous low-Ti lamprophyres and mafic dykes from Shandong in previous studies [14-19](Fig. 2). The trace element patterns reflect either contamination by wall rocks during magma ascend or a mantle source metasomatized recycled crustal rocks. Notably, the studied lamprophyres have higher Ba (up to 243 ppm), Sr (up to 1790 ppm) and $\Sigma$REE (166–362 ppm) contents than those of the average continental crust (Ba=390 ppm; Sr=325 ppm; [20]) and the wall rocks (e.g., $\Sigma$REE = ~30–80 ppm, Sr=300 – 600 μg/g; [19,21]), indicating that the magmas are insensitive to crustal or wallrock contamination. In addition, granites have much lower Mg contents than those of lamprophyres, which has limited influences on Mg isotopic compositions of the latter. Thus, the effect of crustal contamination during magma ascending is limited, and the high $^{87}Sr/^{86}Sr$ (up to 0.71072) and light $\delta^{26}Mg$ (low to -0.59‰) of these lamprophyres are insensitive to wallrock contamination and should reflect isotopic signatures of the primary magmas.

The lamprophyres in this study show linear correlations between MgO and other oxides (Fig. S1), indicating possible fractional crystallization involving olivine and pyroxene during magma evolution. Magnesium isotope fractionation during magma differentiation involving olivine or pyroxene is limited, typically <0.07‰ [22]. The fractional crystallization of plagioclase can be excluded by the absence of Eu anomalies. As shown in Fig. S1, the High-MgO subgroup exhibit weak correlations between different oxides, which is consistent with recycled carbonates in their mantle sources because the addition of recycled carbonates (high MgO, CaO and low Ni contents) can enhance the MgO contents and dilute Ni contents of the basaltic melts [23]. The positive correlation between MgO and Cr (Fig. S1(f)) indicates that lamprophyres may have experienced fractional crystallization of chromite, which is a MgO-rich mineral (MgO>10 wt%) and has heavier $\delta^{26}Mg$ compositions than that of silicate minerals [24], but this

process has not affected the $\delta^{26}$Mg of lamprophyres, which is illustrated by the absence of relationship between $\delta^{26}$Mg and Cr contents (Fig. S2).

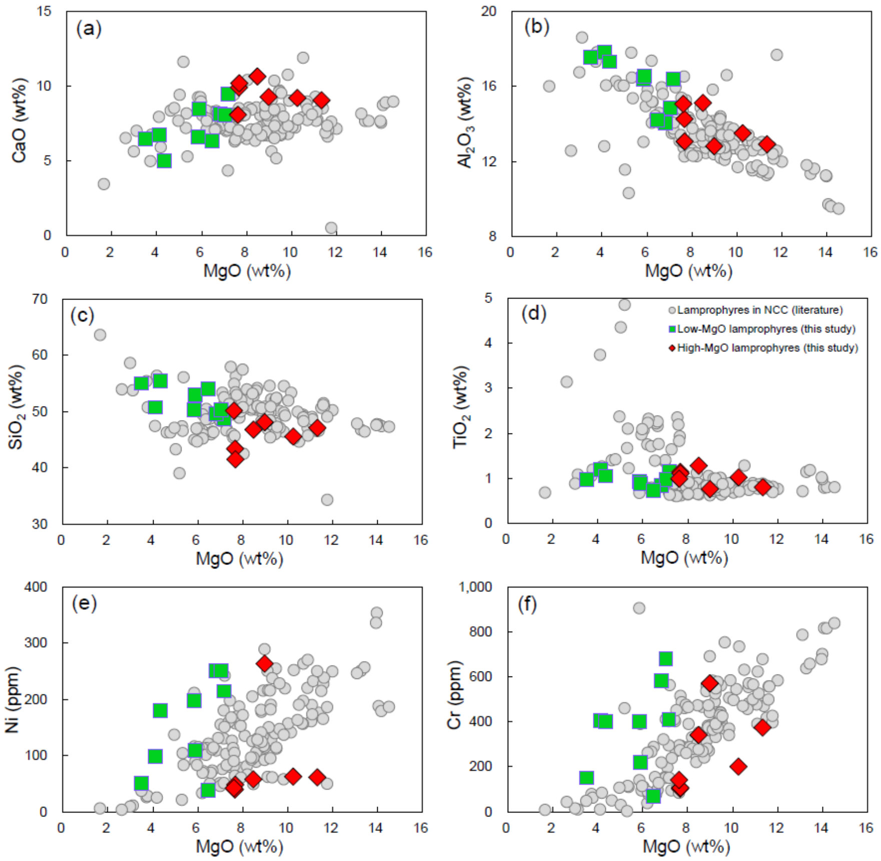


**Figure S1.** Plots of CaO (a), $Al_2O_3$ (b), $SiO_2$ (c), $TiO_2$ (d), Ni (e) and Cr (f) against MgO. The literature data (gray circles) are from Table S2.

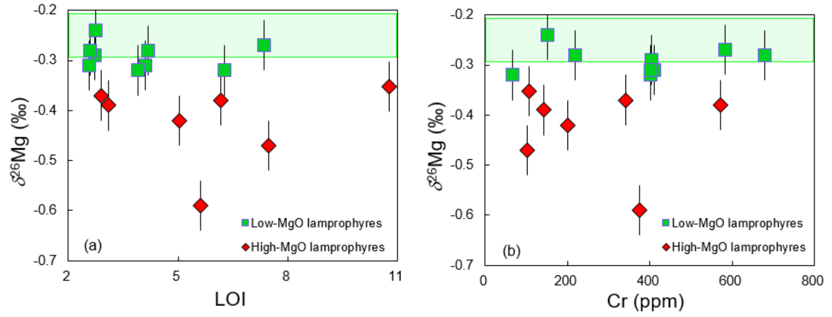


**Figure S2.** Plots of $\delta^{26}$Mg and LOI (a), and Cr (b).

## METHODS FOR CALCULATING $CO_2$ CONTENTS IN MIs

As shown in Fig. 5 and Fig. S3, the large variability in volume and number of bubbles per melt inclusion is the evidence of heterogeneous entrapment of MIs [25]. These bubbles are interpreted as gas exsolution bubbles of a $CO_2$-rich fluid phase, which likely exsolved from silicate melt before or during their entrapment [26]. The single shrinkage bubble per MI was likely formed by the differential contraction of the melt that trapped as MI without gas exsolution bubbles. Other one or more gas exsolution bubbles per MI, which were trapped along with melt, may be able to accommodate the post-entrapment shrinkage. The formation of these bubbles results in a migration of $CO_2$ from the melt to the bubbles and leads to strong enrichment of $CO_2$ in the bubbles, whereas $H_2O$ remains into the melt [25]. Thus, we only measured the $CO_2$ contents of bubbles in this study and estimated that in glass of MIs through previous study [27]. Some bubbles appear to be empty, which may be formed by the leakage or total loss of carbon species possibly due to mechanical micro-fracturing or micro-cracking. The amount of MIs in High-MgO lamprophyres is much larger than that in Low-MgO lamprophyres (Fig. S3) by the observation using reflected and transmitted light optical microscopy. Thus, we mainly analyzed the $CO_2$ contents of MIs in High-MgO lamprophyres in this study. Among about two hundred of MIs analysed in this study, >80% MIs contain vapour bubbles and about 20% vapour bubbles contain $CO_2$. Because of the resonance effect, the presence of $CO_2$ in the bubbles of MIs can be confirmed by the presence of two characteristic peaks, at ~1285 $cm^{-1}$ and ~1388 $cm^{-1}$, defining a Fermi diad in the Raman spectrum [28]. Fermi diads can be observed in the Raman spectra of bubbles down to 1 μm in diameter, thus the success of the technique is not limited by bubble size in this study.

Previous studies have revealed that the spacing of the Fermi diad ($\Delta$ $cm^{-1}$) is related to the fluid density ($d$) and documented several calibrations for measuring the density of pure $CO_2$ fluid inclusions [28-33]. The range of $CO_2$ density determined from 0.1 to 1.24 $g/cm^3$ and the accuracy is better than 5%

[32]. Due to different calibration procedures, discrepancies exist among these studies [32]. The maximum difference in $CO_2$ densities estimated by these different calibrations is about 0.1 g/cm$^3$ at Fermi diad split between ca. 104 and ca. 105 cm$^{-1}$ [28,32]. The range of Fermi diad split ($\Delta$) of bubbles in Shandong lamprophyre melt inclusions is from 102.7 cm$^{-1}$ to 104.5 cm$^{-1}$, with an average of 103.7 cm$^{-1}$, and the differences among these calibrations at this value are small. Compared with other calibrations, the densimeter proposed by Kawakami et al. [29] is valid to lower Fermi diad split. Thus, given the low Δ of $CO_2$-bearing bubbles in Shandong lamprophyre melt inclusions, we select the Kawakami et al. [29] densimeter for estimating the density of $CO_2$-bearing bubbles:

$$d = -0.03238697\Delta^3 + 10.08428\Delta^2 - 1046.189\Delta + 36163.67$$

We assume that the MIs are cuboid and their depth was equal to the width, then the volumes of MIs can be calculated. The bubbles usually have spherical shape and most of them range from 1 to 8 μm in diameter. The volumes of bubble can also be calculated by their diameters. Detailed parameters of MIs and bubbles that were used to calculate the density of $CO_2$-bearing bubbles are presented in Table S6. The uncertainties of $CO_2$ concentrations in the MIs are mainly from the determination of the volume of the bubbles and inclusions, with analysis of larger inclusions and bubbles generating larger accuracy. The inclusion and bubble measurements approximately have errors of ±10%, corresponding to errors of ±20% in the calculated bubble $CO_2$ content.

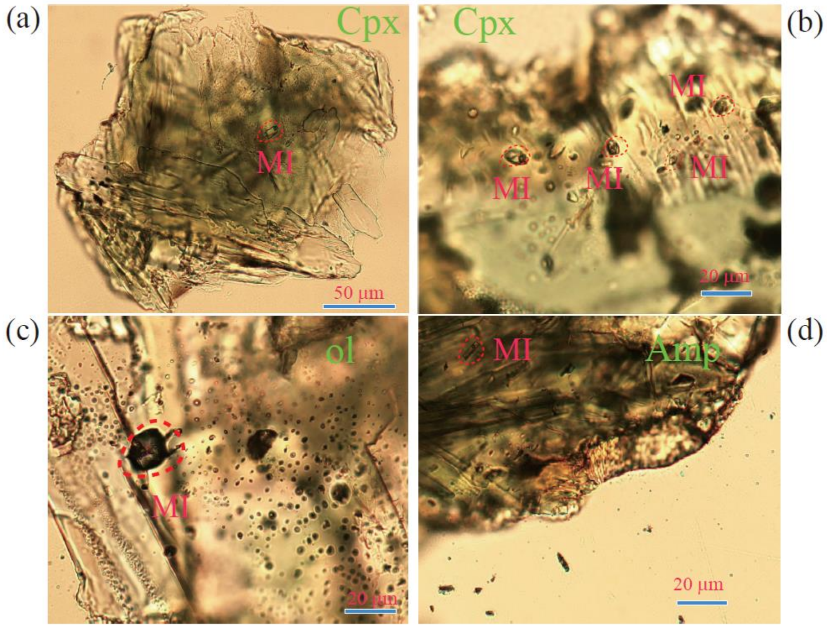


**Figure S3.** Representative bubble-bearing melt inclusions from the Shandong lamprophyres at transmitted light optical microscopy. The red circles indicate the bubble-bearing melt inclusions. (a) Single MI hosted in clinopyxenes (Cpx) of Low-MgO lamprophyre (QX03), (b) Multi MIs hosted in clinopyxenes of High-MgO lamprophyre (HY11). (c) MIs hosted in olivine (Ol), (d) MIs hosted in amphibole (Amp).

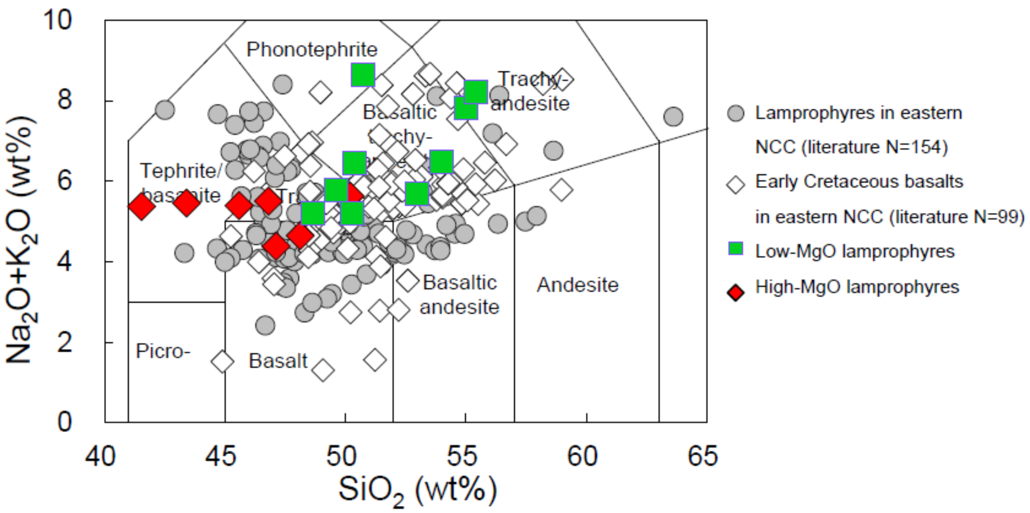


**Figure S4.** Plots of total alkali ($Na_2O+K_2O$) vs. $SiO_2$ for the lamprophyres and early Cretaceous volcanic rocks in the NCC (all data are listed in Tables S1-S2).

**Table S1.** Major and trace element contents of Shandong lamprophyres in this study.

| Sample No. | High-MgO lamprophyres | | | | | | | Low-MgO lamprophyres | | | | | | | | |
|---|---|---|---|---|---|---|---|---|---|---|---|---|---|---|---|---|
| | HY11 | QX01 | LL06 | PD03 | HY04 | HY12 | QX04 | LZ04 | PD08 | LL01 | HY06 | HY03 | LZ02 | QX03 | LL04 | PD04 |
| Latitude | | 37.2653 | 37.4539 | 36.8719 | | | 37.1761 | 37.1692 | 36.8719 | 37.4539 | | | 37.1692 | 37.1761 | 37.4539 | 36.8719 |
| Longitude | | 120.880 | 120.505 | 120.014 | | | 121.376 | 120.155 | 120.014 | 120.505 | | | 120.155 | 121.376 | 120.505 | 120.014 |
| *Major elements (wt%)* | | | | | | | | | | | | | | | | |
| $SiO_2$ | 43.4 | 45.6 | 46.8 | 47.1 | 48.1 | 41.5 | 50.2 | 48.6 | 49.6 | 50.3 | 50.4 | 50.8 | 53.0 | 54.0 | 55.0 | 55.4 |
| $Al_2O_3$ | 14.3 | 13.5 | 15.1 | 12.9 | 12.8 | 13.1 | 15.1 | 16.4 | 14.1 | 14.8 | 16.4 | 17.8 | 16.6 | 14.2 | 17.6 | 17.3 |
| $Fe_2O_3^T$ | 9.18 | 8.36 | 9.38 | 8.80 | 9.26 | 8.77 | 7.90 | 7.97 | 8.15 | 7.88 | 7.03 | 7.88 | 7.33 | 6.41 | 5.99 | 6.26 |
| CaO | 9.90 | 9.20 | 10.64 | 9.05 | 9.27 | 10.18 | 8.08 | 9.47 | 8.15 | 8.05 | 6.62 | 6.75 | 8.44 | 6.32 | 6.49 | 4.98 |
| MgO | 7.66 | 10.25 | 8.48 | 11.33 | 8.98 | 7.67 | 7.61 | 7.16 | 6.83 | 7.02 | 5.85 | 4.12 | 5.89 | 6.47 | 3.49 | 4.34 |
| $K_2O$ | 2.81 | 3.05 | 2.57 | 2.28 | 2.20 | 3.41 | 2.98 | 1.95 | 3.31 | 2.57 | 2.89 | 4.60 | 2.27 | 2.94 | 3.48 | 4.02 |
| $Na_2O$ | 2.64 | 2.34 | 2.93 | 2.10 | 2.44 | 1.96 | 2.65 | 3.23 | 2.45 | 2.63 | 3.55 | 4.03 | 3.40 | 3.53 | 4.34 | 4.20 |
| $P_2O_5$ | 0.89 | 0.71 | 0.62 | 0.30 | 0.41 | 0.84 | 0.78 | 0.54 | 0.47 | 0.47 | 0.49 | 1.16 | 0.40 | 0.44 | 0.59 | 0.83 |
| MnO | 0.15 | 0.13 | 0.15 | 0.13 | 0.14 | 0.15 | 0.12 | 0.13 | 0.11 | 0.14 | 0.14 | 0.14 | 0.12 | 0.11 | 0.10 | 0.12 |
| $TiO_2$ | 1.14 | 1.02 | 1.28 | 0.80 | 0.76 | 1.11 | 0.99 | 1.16 | 0.83 | 0.97 | 0.92 | 1.19 | 0.89 | 0.73 | 0.98 | 1.05 |
| LOI | 7.48 | 5.03 | 2.90 | 5.61 | 6.17 | 10.77 | 3.10 | 4.10 | 7.35 | 4.18 | 6.27 | 2.72 | 2.61 | 3.91 | 2.76 | 2.58 |
| *Trace elements (ppm)* | | | | | | | | | | | | | | | | |
| La | 51.7 | 56.1 | 47.0 | 50.5 | 66.2 | 52.0 | 77.6 | 55.8 | 45.3 | 59.9 | 50.9 | 52.3 | 62.6 | 47.1 | 36.6 | 53.5 |
| Ce | 109 | 112 | 97.7 | 103 | 155 | 110 | 164 | 105 | 90.1 | 117 | 112 | 98.6 | 126 | 90.3 | 74.5 | 101 |
| Pr | 13.2 | 12.8 | 10.7 | 12.0 | 14.2 | 13.2 | 18.8 | 9.77 | 9.82 | 12.7 | 10.5 | 11.6 | 12.6 | 9.97 | 7.96 | 10.4 |
| Nd | 50.4 | 47.4 | 42.4 | 46.9 | 51.9 | 50.9 | 70.1 | 35.2 | 36.7 | 47.4 | 37.1 | 41.3 | 48.2 | 35.6 | 29.8 | 39.2 |
| Sm | 8.68 | 7.31 | 6.82 | 7.70 | 7.72 | 8.69 | 10.42 | 6.76 | 5.79 | 7.31 | 5.75 | 6.77 | 7.09 | 5.35 | 4.67 | 5.91 |
| Eu | 2.77 | 2.37 | 1.88 | 2.23 | 2.04 | 3.01 | 3.09 | 1.77 | 1.56 | 1.94 | 1.57 | 2.22 | 2.08 | 1.84 | 1.51 | 1.68 |
| Gd | 6.98 | 5.43 | 5.64 | 6.25 | 6.10 | 7.70 | 8.86 | 6.40 | 4.28 | 5.69 | 4.81 | 5.25 | 5.74 | 4.85 | 4.03 | 4.78 |

Continued

| | | | | | | | | | | | | | | | | |
|---|---|---|---|---|---|---|---|---|---|---|---|---|---|---|---|---|
| Tb | 0.97 | 0.81 | 0.70 | 0.87 | 0.61 | 0.98 | 1.02 | 0.70 | 0.49 | 0.56 | 0.51 | 0.47 | 0.60 | 0.61 | 0.53 | 0.50 |
| Dy | 4.22 | 3.58 | 3.79 | 4.55 | 3.48 | 4.21 | 4.04 | 3.44 | 3.28 | 3.29 | 3.09 | 2.12 | 3.60 | 2.53 | 2.72 | 3.09 |
| Ho | 0.76 | 0.67 | 0.67 | 0.84 | 0.68 | 0.77 | 0.73 | 0.67 | 0.62 | 0.66 | 0.64 | 0.77 | 0.66 | 0.49 | 0.48 | 0.57 |
| Er | 1.99 | 1.77 | 1.98 | 2.50 | 2.05 | 1.97 | 1.87 | 1.77 | 1.91 | 1.87 | 1.83 | 1.82 | 1.94 | 1.30 | 1.44 | 1.75 |
| Tm | 0.27 | 0.25 | 0.28 | 0.39 | 0.24 | 0.27 | 0.27 | 0.34 | 0.25 | 0.23 | 0.24 | 0.41 | 0.25 | 0.19 | 0.20 | 0.22 |
| Yb | 1.64 | 1.52 | 1.80 | 2.44 | 1.66 | 1.63 | 1.61 | 1.44 | 1.43 | 1.53 | 1.57 | 1.21 | 1.64 | 1.14 | 1.31 | 1.52 |
| Lu | 0.21 | 0.18 | 0.27 | 0.38 | 0.23 | 0.21 | 0.21 | 0.21 | 0.24 | 0.22 | 0.21 | 0.17 | 0.21 | 0.14 | 0.19 | 0.20 |
| Rb | 26.71 | 28.6 | 37.8 | 55.5 | 64.9 | 48.1 | 51.5 | 73.4 | 115 | 71.8 | 50.9 | 52.4 | 82.8 | 29.2 | 174 | 72.9 |
| Ba | 1709 | 1933 | 1821 | 365 | 1560 | 2943 | 1699 | 1433 | 1365 | 1781 | 1208 | 786 | 2258 | 1669 | 224 | 2290 |
| Th | 4.42 | 4.38 | 9.34 | 10.1 | 10.1 | 4.30 | 7.71 | 9.05 | 8.20 | 9.21 | 8.49 | 8.91 | 9.54 | 6.32 | 6.03 | 8.95 |
| U | 1.28 | 1.25 | 1.41 | 2.08 | 1.79 | 1.32 | 1.60 | 1.44 | 1.42 | 1.62 | 1.44 | 1.32 | 1.65 | 1.94 | 1.30 | 1.84 |
| Nb | 7.59 | 5.62 | 4.78 | 5.25 | 4.78 | 8.08 | 6.99 | 6.32 | 6.79 | 4.32 | 6.09 | 5.32 | 7.04 | 5.61 | 5.16 | 5.48 |
| Ta | 0.47 | 0.32 | 0.37 | 0.34 | 0.45 | 0.48 | 0.60 | 0.44 | 0.63 | 0.31 | 0.47 | 0.57 | 0.45 | 0.43 | 0.33 | 0.39 |
| Pb | 16.45 | 16.9 | 13.6 | 11.1 | 34.1 | 13.3 | 12.3 | 34.7 | 21.1 | 29.7 | 31.3 | 30.7 | 61.9 | 14.9 | 8.5 | 33.6 |
| Sr | 1084 | 947 | 1590 | 357 | 1358 | 1529 | 1043 | 1233 | 397 | 1790 | 721 | 926 | 960 | 863 | 329 | 1159 |
| Zr | 161 | 142 | 126 | 139 | 137 | 171 | 152 | 150 | 149 | 140 | 155 | 161 | 165 | 134 | 144 | 187 |
| Hf | 4.22 | 3.66 | 3.44 | 3.86 | 3.07 | 4.35 | 4.02 | 3.51 | 3.76 | 3.21 | 3.37 | 4.32 | 3.49 | 3.46 | 3.89 | 4.08 |
| Y | 16.03 | 13.4 | 18.2 | 22.5 | 17.0 | 16.0 | 16.6 | 15.0 | 15.8 | 15.9 | 15.6 | 16.9 | 17.2 | 10.1 | 14.3 | 15.8 |
| V | 60.5 | 51.1 | 184 | 211 | 182 | 59.4 | 59.1 | 158 | 152 | 190 | 144 | 147 | 153 | 29.2 | 117 | 153 |
| Cr | 103.2 | 201 | 341 | 374 | 571 | 106 | 142 | 411 | 582 | 678 | 402 | 405 | 219 | 67.4 | 151 | 402 |
| Co | 11.19 | 12.4 | 42.9 | 50.8 | 41.3 | 13.2 | 8.26 | 29.8 | 39.1 | 42.4 | 33.1 | 32.1 | 28.4 | 13.7 | 26.0 | 33.0 |
| Ni | 39.4 | 62.4 | 57.6 | 61.0 | 263 | 48.1 | 42.0 | 214 | 250 | 250 | 197 | 99.0 | 109 | 38.6 | 51.0 | 180 |

**Table S2**. Major elements and Sr-Nd isotopic compositions of lamprophyres in eastern NCC and Cenozoic basalts in Shandong

| Sample No. | $SiO_2$ | $TiO_2$ | $Al_2O_3$ | $TFe_2O_3$ | MnO | MgO | CaO | $Na_2O$ | $K_2O$ | $P_2O_5$ | Total | $^{87}Sr/^{86}Sr(i)$ | $\varepsilon_{Nd}(t)$ | Reference |
|---|---|---|---|---|---|---|---|---|---|---|---|---|---|---|
| *Lamprophyres in eastern NCC* | | | | | | | | | | | | | | |
| LKXDJ-3-1 | 47.7 | 0.79 | 9.7 | 7.44 | 0.08 | 14.1 | 8.73 | 1.71 | 1.88 | 0.47 | 99.8 | 0.70893 | -15.4 | [34] |
| LKXDJ-3-3 | 47.4 | 0.80 | 9.6 | 7.39 | 0.08 | 14.2 | 8.90 | 1.64 | 1.84 | 0.50 | 99.8 | 0.70887 | -15.6 | |
| LKXDJ-3-4 | 47.3 | 0.80 | 9.5 | 7.43 | 0.08 | 14.5 | 8.96 | 1.56 | 1.84 | 0.49 | 99.8 | 0.70893 | -15.4 | |
| LKXDJ-6-1 | 54.5 | 0.73 | 12.7 | 7.30 | 0.10 | 9.84 | 6.80 | 2.54 | 2.16 | 0.41 | 99.8 | 0.70986 | -16.7 | |
| LKXDJ-6-3 | 54.6 | 0.70 | 13.1 | 7.09 | 0.09 | 9.20 | 6.55 | 2.84 | 2.10 | 0.39 | 99.8 | 0.70982 | -16.4 | |
| LKXDJ-6-4 | 53.4 | 0.73 | 12.6 | 7.38 | 0.10 | 10.2 | 7.23 | 2.46 | 1.97 | 0.40 | 99.8 | 0.70986 | -16.6 | |
| LKXDJ-6-5 | 54.2 | 0.72 | 13.1 | 6.96 | 0.09 | 8.65 | 7.14 | 2.72 | 2.19 | 0.38 | 99.8 | 0.70983 | -16.5 | |
| LKXDJ-7-1 | 56.3 | 0.62 | 12.8 | 5.98 | 0.08 | 7.70 | 6.60 | 2.81 | 2.13 | 0.23 | 99.8 | 0.70941 | -18.2 | |
| LKXDJ-7-2 | 57.5 | 0.64 | 12.6 | 6.11 | 0.08 | 8.00 | 6.47 | 2.82 | 2.18 | 0.23 | 99.8 | 0.70927 | -18.5 | |
| LKXDJ-7-3 | 57.9 | 0.63 | 13.1 | 6.03 | 0.09 | 7.47 | 6.30 | 2.99 | 2.15 | 0.23 | 99.8 | 0.70925 | -18.2 | |
| LKXDJ-9-1 | 54.9 | 0.70 | 12.8 | 5.92 | 0.07 | 7.62 | 7.06 | 2.27 | 2.42 | 0.26 | 99.8 | 0.70939 | -17.6 | |
| LKXDJ-9-2 | 53.7 | 0.72 | 12.5 | 6.13 | 0.08 | 8.61 | 7.24 | 2.09 | 2.20 | 0.24 | 99.8 | 0.70945 | -17.8 | |
| LKXDJ-9-3 | 53.9 | 0.68 | 12.5 | 5.99 | 0.08 | 8.19 | 7.10 | 2.06 | 2.31 | 0.26 | 99.8 | 0.70943 | -17.7 | |
| LKXDJ-10-1 | 46.5 | 0.88 | 11.3 | 8.05 | 0.12 | 11.3 | 8.90 | 2.04 | 2.79 | 0.70 | 99.8 | 0.70952 | -17.1 | |
| LKXDJ-10-2 | 46.6 | 0.85 | 11.4 | 8.17 | 0.11 | 11.4 | 8.51 | 2.09 | 2.90 | 0.66 | 99.8 | 0.70952 | -17.2 | |
| ZYLL-5-1 | 48.3 | 0.79 | 11.4 | 7.41 | 0.18 | 11.1 | 9.28 | 1.00 | 1.74 | 0.31 | 99.8 | 0.70995 | -16.7 | |
| ZYLL-5-2 | 49.4 | 0.78 | 11.5 | 7.26 | 0.16 | 10.7 | 8.96 | 1.03 | 2.17 | 0.32 | 99.8 | 0.70985 | -16.7 | |
| ZYLL-5-3 | 49.2 | 0.79 | 11.7 | 7.43 | 0.17 | 10.7 | 8.78 | 1.09 | 2.00 | 0.33 | 99.8 | 0.70992 | -16.9 | |
| ZYJL-1-1 | 47.1 | 0.89 | 11.8 | 7.44 | 0.10 | 10.9 | 7.84 | 1.50 | 2.61 | 0.42 | 99.8 | 0.70903 | -16.2 | |
| ZYJL-1-2 | 47.8 | 0.86 | 11.5 | 7.66 | 0.10 | 11.2 | 7.74 | 1.67 | 2.47 | 0.42 | 99.8 | 0.70900 | -16.6 | |
| LZYD-1-6 | 49.7 | 0.90 | 13.0 | 7.91 | 0.12 | 8.89 | 8.24 | 2.08 | 2.58 | 0.95 | 99.8 | 0.71092 | -17.0 | |
| LZYD-1-7 | 50.4 | 0.84 | 12.7 | 7.57 | 0.11 | 8.61 | 8.05 | 2.18 | 2.56 | 0.88 | 99.8 | 0.71094 | -17.5 | |
| LZYD-1-8 | 49.3 | 0.87 | 12.4 | 7.73 | 0.12 | 9.03 | 8.65 | 1.86 | 2.79 | 0.91 | 99.8 | 0.71099 | -17.2 | |

Continued

| | | | | | | | | | | | | | |
|---|---|---|---|---|---|---|---|---|---|---|---|---|---|
| LZYD-1-9 | 50.2 | 0.85 | 12.7 | 7.72 | 0.13 | 9.00 | 8.24 | 2.01 | 3.05 | 0.92 | 99.8 | 0.71090 | -17.4 |
| LZYD-1-10 | 50.0 | 0.86 | 12.4 | 7.79 | 0.13 | 8.97 | 8.67 | 1.99 | 3.48 | 0.95 | 99.8 | 0.71092 | -17.1 |
| LZYD-4-1 | 53.3 | 0.99 | 13.5 | 6.64 | 0.10 | 7.10 | 7.00 | 2.51 | 3.83 | 0.64 | 99.8 | 0.70919 | -14.3 |
| LZYD-4-2 | 52.8 | 1.12 | 13.5 | 7.23 | 0.11 | 8.40 | 6.84 | 2.39 | 3.51 | 0.68 | 99.8 | 0.70932 | -13.9 |
| PDCZ-1-1 | 47.7 | 0.98 | 11.2 | 8.95 | 0.11 | 14.0 | 7.55 | 1.97 | 4.33 | 0.87 | 99.8 | 0.70979 | -17.2 |
| PDCZ-1-2 | 47.6 | 1.02 | 11.3 | 8.86 | 0.12 | 14.0 | 7.66 | 2.03 | 4.21 | 0.94 | 99.8 | 0.70980 | -17.1 |
| PDCZ-2-1 | 46.8 | 1.14 | 11.3 | 9.28 | 0.11 | 13.3 | 7.67 | 1.69 | 3.55 | 0.73 | 99.9 | 0.70917 | -14.9 |
| PDCZ-2-2 | 46.4 | 1.18 | 11.6 | 9.41 | 0.11 | 13.4 | 7.67 | 1.79 | 3.44 | 0.74 | 99.8 | 0.70912 | -14.6 |
| LXHL-1-1 | 48.4 | 1.37 | 14.7 | 9.25 | 0.17 | 6.98 | 8.19 | 2.66 | 3.05 | 0.82 | 99.8 | 0.70966 | -15.0 |
| LXHL-1-2 | 48.4 | 1.40 | 14.7 | 9.29 | 0.16 | 7.00 | 8.16 | 2.58 | 3.05 | 0.81 | 99.8 | 0.70968 | -15.1 |
| LXHL-1-3 | 48.7 | 1.39 | 14.6 | 9.41 | 0.16 | 6.54 | 8.29 | 2.59 | 3.13 | 0.78 | 99.8 | 0.70965 | -14.8 |
| MPYL-1-1 | 52.0 | 0.68 | 11.7 | 7.01 | 0.09 | 10.0 | 7.28 | 2.30 | 1.88 | 0.25 | 99.8 | 0.70930 | -16.2 |
| MPYL-1-2 | 52.2 | 0.71 | 12.2 | 6.91 | 0.09 | 9.27 | 7.19 | 2.49 | 1.87 | 0.27 | 99.8 | 0.70957 | -16.1 |
| MPYL-1-3 | 52.0 | 0.71 | 12.1 | 6.88 | 0.09 | 9.45 | 7.23 | 2.35 | 1.89 | 0.28 | 99.8 | 0.70944 | -16.0 |
| MPYL-2-1 | 52.4 | 0.64 | 12.4 | 6.44 | 0.09 | 9.51 | 6.71 | 2.50 | 1.72 | 0.21 | 99.8 | 0.70873 | -15.5 |
| MPYL-2-2 | 52.4 | 0.64 | 12.4 | 6.47 | 0.09 | 9.50 | 6.79 | 2.43 | 1.75 | 0.21 | 99.8 | 0.70858 | -15.6 |
| MPYL-2-3 | 52.8 | 0.64 | 13.1 | 6.26 | 0.09 | 8.49 | 6.77 | 2.58 | 2.10 | 0.23 | 99.9 | 0.70859 | -15.6 |
| MPWGZ-1-1 | 50.2 | 0.76 | 12.0 | 7.58 | 0.13 | 12.0 | 7.15 | 1.95 | 1.49 | 0.26 | 99.8 | 0.70969 | -15.4 |
| MPWGZ-1-3 | 51.5 | 0.76 | 12.6 | 7.51 | 0.11 | 11.6 | 6.63 | 2.16 | 1.75 | 0.27 | 99.8 | 0.70977 | -15.6 |
| MPWGZ-1-5 | 50.9 | 0.77 | 12.2 | 7.50 | 0.13 | 11.6 | 7.08 | 2.14 | 1.55 | 0.26 | 99.8 | 0.70966 | -15.7 |
| LYGC-2-1 | 50.7 | 0.83 | 12.8 | 7.96 | 0.11 | 11.5 | 6.89 | 2.58 | 2.42 | 0.52 | 99.8 | | |
| LYGC-2-3 | 49.0 | 0.80 | 12.6 | 7.86 | 0.10 | 11.1 | 7.68 | 2.71 | 2.43 | 0.47 | 99.8 | 0.70845 | -12.6 |
| LYGC-2-4 | 49.1 | 0.81 | 12.6 | 7.74 | | 11.8 | 7.62 | 2.41 | 2.51 | 0.50 | 99.8 | 0.70839 | -15.3 |
| RSXC-2-1 | 50.1 | 0.83 | 13.8 | 7.58 | | 9.64 | 7.21 | 2.49 | 2.25 | 0.43 | 99.8 | 0.70921 | -14.6 |
| RSXC-2-2 | 50.3 | 0.83 | 14.9 | 7.23 | | 8.38 | 6.35 | 2.94 | 2.89 | 0.47 | 99.9 | 0.70858 | -14.5 |
| RSXC-2-5 | 49.8 | 0.82 | 13.7 | 7.53 | | 9.84 | 7.34 | 2.52 | 2.18 | 0.43 | 99.8 | 0.70868 | -14.4 |

Continued

| | | | | | | | | | | | | | | |
|---|---|---|---|---|---|---|---|---|---|---|---|---|---|---|
| RSXC-3-1 | 49.7 | 0.84 | 13.6 | 7.51 | | 9.17 | 7.45 | 2.57 | 2.58 | 0.50 | 99.8 | 0.70855 | -14.9 | |
| RSXC-3-2 | 49.7 | 0.84 | 13.4 | 7.53 | | 8.91 | 7.62 | 2.64 | 2.56 | 0.51 | 99.8 | 0.70855 | -14.9 | |
| RSXC-3-4 | 50.4 | 0.85 | 14.2 | 7.39 | | 8.94 | 6.70 | 2.59 | 2.67 | 0.51 | 99.8 | 0.70908 | -14.8 | |
| WDSC-1-1 | 50.6 | 0.90 | 13.3 | 7.03 | | 8.10 | 8.55 | 2.35 | 1.97 | 0.55 | 99.8 | 0.70803 | -13.5 | |
| WDSC-1-2 | 50.7 | 0.89 | 13.4 | 7.11 | | 8.44 | 8.57 | 2.33 | 2.02 | 0.57 | 99.8 | 0.70806 | -13.4 | |
| WDSC-1-5 | 50.7 | 0.86 | 13.3 | 7.10 | | 8.83 | 8.37 | 2.30 | 2.07 | 0.57 | 99.8 | 0.70805 | -13.4 | |
| WDSC-2-1 | 50.7 | 0.80 | 14.1 | 7.79 | | 8.73 | 6.70 | 2.56 | 3.08 | 0.58 | 99.8 | 0.70814 | -13.4 | |
| WDSC-2-2 | 50.8 | 0.79 | 13.9 | 7.70 | 0.12 | 8.86 | 6.96 | 2.17 | 3.51 | 0.59 | 99.8 | 0.70814 | -13.4 | |
| WDSC-2-3 | 51.0 | 0.76 | 14.2 | 7.60 | 0.12 | 8.66 | 6.72 | 2.10 | 3.82 | 0.56 | 99.8 | 0.70811 | -13.5 | |
| WDSC-3-1 | 52.0 | 0.83 | 12.3 | 7.17 | 0.12 | 8.21 | 8.42 | 1.77 | 2.54 | 0.44 | 99.8 | 0.70822 | -8.9 | |
| WDSC-3-3 | 51.8 | 0.86 | 12.3 | 7.14 | 0.14 | 7.66 | 8.52 | 1.77 | 2.70 | 0.44 | 99.8 | 0.70832 | -9.1 | |
| WDSC-3-4 | 52.1 | 0.84 | 12.4 | 7.13 | 0.12 | 7.93 | 8.19 | 1.82 | 2.49 | 0.44 | 99.8 | 0.70796 | -9.0 | |
| JJLT-01 | 51.2 | 0.79 | 12.4 | 8.47 | 0.14 | 11.6 | 7.35 | 1.57 | 3.23 | 0.41 | 99.8 | 0.70903 | -14.9 | [15] |
| JJLT-02 | 50.0 | 0.82 | 13.1 | 8.05 | 0.15 | 10.5 | 7.89 | 1.68 | 3.09 | 0.45 | 99.7 | 0.70953 | -14.2 | |
| JJLT-03 | 50.8 | 0.62 | 13.2 | 7.84 | 0.16 | 8.98 | 7.70 | 1.51 | 3.55 | 0.37 | 99.7 | 0.70934 | -15.5 | |
| JJLT-04 | 47.6 | 0.97 | 14.4 | 10.7 | 0.30 | 9.26 | 8.34 | 1.65 | 3.13 | 0.36 | 99.9 | 0.70969 | -14.6 | |
| JJLT-05 | 47.9 | 0.71 | 11.8 | 9.14 | 0.15 | 13.1 | 8.15 | 1.00 | 3.01 | 0.30 | 99.9 | 0.70922 | -14.3 | |
| JJLT-06 | 48.6 | 0.77 | 13.0 | 8.93 | 0.16 | 11.1 | 8.29 | 1.09 | 3.09 | 0.32 | 99.8 | 0.70960 | -13.9 | |
| JJLT-07 | 49.0 | 0.82 | 13.0 | 8.34 | 0.16 | 10.7 | 7.73 | 1.67 | 3.10 | 0.39 | 99.9 | 0.70932 | -14.8 | |
| JJHT-01 | 46.6 | 2.1 | 16.5 | 9.80 | 0.14 | 5.33 | 8.17 | 4.03 | 2.85 | 0.96 | 99.7 | 0.70683 | 0.9 | |
| JJHT-02 | 46.4 | 2.16 | 14.3 | 11.8 | 0.20 | 6.65 | 7.11 | 3.79 | 2.84 | 0.84 | 99.9 | 0.70573 | 1.8 | |
| JJHT-03 | 46.0 | 2.22 | 14.1 | 12.1 | 0.21 | 6.59 | 7.07 | 3.55 | 3.10 | 0.91 | 99.9 | | | |
| JJHT-04 | 47.1 | 2.07 | 15.8 | 10.6 | 0.15 | 6.03 | 8.04 | 3.19 | 2.89 | 1.00 | 99.9 | 0.70720 | 0.1 | |
| JJHT-05 | 46.4 | 2.26 | 16.0 | 11.3 | 0.20 | 6.65 | 8.33 | 3.74 | 2.84 | 0.89 | 100 | 0.70571 | 0.2 | |
| JJHT-06 | 45.9 | 2.32 | 15.2 | 12.1 | 0.21 | 6.25 | 7.82 | 3.48 | 3.29 | 1.02 | 100 | 0.70546 | 0.9 | |
| JJHT-07 | 47.0 | 1.97 | 15.0 | 10.6 | 0.18 | 5.94 | 9.25 | 2.40 | 2.89 | 0.95 | 100 | 0.70571 | -0.8 | |

Continued

| | | | | | | | | | | | | | |
|---|---|---|---|---|---|---|---|---|---|---|---|---|---|
| JJHT-08 | 46.0 | 2.35 | 15.4 | 11.6 | 0.20 | 7.53 | 8.16 | 4.04 | 2.75 | 0.72 | 100 | 0.70640 | 0.4 | |
| JJHT-09 | 45.2 | 2.23 | 15.2 | 11.1 | 0.19 | 6.22 | 7.68 | 4.03 | 2.69 | 1.12 | 99.8 | 0.70553 | 1.5 | |
| JJHT-10 | 45.4 | 2.19 | 14.7 | 11.0 | 0.19 | 7.58 | 8.10 | 3.76 | 2.52 | 0.85 | 99.7 | | | |
| LM-1 | 46.4 | 1.76 | 15.6 | 10.5 | 0.17 | 6.7 | 8.20 | 3.10 | 2.51 | 0.70 | 99.7 | 0.70691 | -0.2 | [16] |
| LM-2 | 45.7 | 1.93 | 16.5 | 11.2 | 0.21 | 7.64 | 7.04 | 3.09 | 2.54 | 0.74 | 100 | | | |
| LM-3 | 50.1 | 1.83 | 13.0 | 9.54 | 0.24 | 7.61 | 7.94 | 3.30 | 2.22 | 0.89 | 99.8 | 0.70845 | 0.2 | |
| LM-4 | 47.1 | 2.37 | 16.1 | 11.2 | 0.30 | 4.96 | 7.68 | 3.62 | 2.78 | 0.94 | 99.8 | 0.70622 | 2.6 | |
| LM-5 | 44.7 | 2.09 | 15.5 | 10.7 | 0.18 | 5.97 | 8.96 | 4.62 | 3.04 | 1.01 | 100 | 0.70708 | 1.3 | |
| LL-01 | 53.2 | 0.65 | 14.5 | 8.13 | 0.11 | 8.44 | 7.13 | 3.24 | 2.50 | 0.06 | 99.9 | 0.70974 | -16.8 | [35] |
| LL-02 | 48.9 | 0.79 | 14.4 | 9.82 | 0.14 | 7.85 | 8.04 | 2.05 | 2.42 | 0.35 | 98.9 | | | |
| LL-03 | 49.3 | 0.79 | 13.2 | 8.87 | 0.12 | 9.89 | 9.02 | 2.30 | 1.94 | 0.04 | 99.9 | 0.71031 | -15.1 | |
| LL-04 | 47.1 | 0.80 | 12.9 | 8.81 | 0.13 | 11.3 | 9.05 | 2.10 | 2.28 | 0.30 | 99.9 | 0.70926 | -13.0 | |
| LL-05 | 48.3 | 0.74 | 12.6 | 8.93 | 0.12 | 10.7 | 8.45 | 2.14 | 2.34 | 0.31 | 99.9 | | | |
| LL-06 | 48.1 | 0.76 | 12.8 | 9.26 | 0.14 | 8.98 | 9.27 | 2.44 | 2.20 | 0.41 | 100 | 0.71006 | -16.2 | |
| LL-07 | 51.0 | 0.66 | 13.6 | 8.29 | 0.11 | 7.20 | 7.55 | 2.26 | 2.99 | 0.22 | 99.7 | 0.71024 | -16.7 | |
| LL-08 | 49.6 | 0.61 | 13.1 | 8.01 | 0.11 | 7.44 | 9.42 | 2.76 | 2.24 | 0.29 | 99.9 | 0.70939 | -16.5 | |
| LL-09 | 47.9 | 0.74 | 13.0 | 9.25 | 0.12 | 9.48 | 8.45 | 2.20 | 2.18 | 0.34 | 100 | | | |
| LL-10 | 51.2 | 0.68 | 13.8 | 8.09 | 0.10 | 7.16 | 7.07 | 2.41 | 2.62 | 0.28 | 99.8 | | | |
| LL-11 | 49.6 | 0.83 | 14.1 | 8.15 | 0.11 | 6.83 | 7.19 | 2.45 | 3.31 | 0.47 | 99.8 | | | |
| PL-01 | 52.5 | 0.70 | 16.3 | 8.86 | 0.08 | 7.17 | 4.35 | 1.24 | 3.54 | 0.23 | 98.9 | 0.70957 | -18.2 | |
| PL-02 | 49.9 | 0.78 | 13.3 | 8.48 | 0.10 | 9.24 | 6.52 | 1.98 | 2.26 | 0.27 | 98.6 | 0.70987 | -14.9 | |
| PL-03 | 46.9 | 0.90 | 13.0 | 10.0 | 0.14 | 9.12 | 9.10 | 1.34 | 3.14 | 0.32 | 99.8 | | | |
| PL-04 | 46.4 | 0.89 | 12.9 | 10.1 | 0.15 | 9.83 | 10.8 | 2.02 | 2.06 | 0.30 | 99.9 | 0.70913 | -13.1 | |
| PL-05 | 50.0 | 0.72 | 13.1 | 7.93 | 0.11 | 9.11 | 5.63 | 2.29 | 2.78 | 0.27 | 98.6 | 0.71003 | -16.2 | |
| PL-06 | 46.9 | 0.93 | 16.4 | 8.32 | 0.13 | 7.29 | 7.24 | 1.44 | 3.49 | 0.58 | 99.9 | 0.70942 | -14.8 | |
| PL-07 | 47.5 | 0.90 | 13.3 | 12.3 | 0.09 | 9.32 | 5.17 | 1.30 | 2.05 | 0.30 | 100 | | | |
| FY306-1 | 46.3 | 1.40 | 16.3 | 8.82 | 0.13 | 4.62 | 8.36 | 3.09 | 1.62 | 0.60 | 99.3 | 0.70761 | -9.5 | [36] |

Continued

| | | | | | | | | | | | | | | |
|---|---|---|---|---|---|---|---|---|---|---|---|---|---|---|
| FY306-2 | 46.3 | 1.42 | 16.1 | 7.71 | 0.126 | 4.78 | 8.53 | 3.04 | 1.62 | 0.60 | 98.6 | | | |
| FY502-1 | 47.4 | 3.74 | 12.8 | 15.4 | 0.23 | 4.10 | 7.94 | 2.92 | 1.16 | 0.65 | 100 | 0.70699 | -10.8 | |
| FY502-2 | 39.0 | 4.85 | 10.3 | 18.6 | 0.20 | 5.19 | 11.6 | 1.02 | 0.38 | 0.71 | 98.7 | 0.70654 | -11.5 | |
| FY502-3 | 43.3 | 4.35 | 11.6 | 16.0 | 0.18 | 5.02 | 9.41 | 3.62 | 0.60 | 1.04 | 99.5 | 0.70608 | -12.5 | |
| FY502-4 | | | | | | | | | | | | 0.70596 | -8.8 | |
| 97RS-3 | 55.4 | 1.05 | 17.3 | 2.06 | 0.12 | 3.72 | 4.98 | 4.02 | 4.20 | 0.83 | 100 | 0.70868 | -14.7 | [37] |
| 97RS-7 | 47.9 | 1.05 | 15.8 | 1.42 | 0.13 | 8.92 | 8.05 | 2.11 | 3.09 | 0.52 | 98.9 | 0.70827 | -13.5 | |
| 97RS-9 | 53.8 | 1.08 | 18.6 | 2.48 | 0.14 | 3.11 | 7.01 | 3.95 | 4.16 | 0.6 | 100 | 0.70788 | -13.2 | |
| 97RS-16 | 50.4 | 0.92 | 16.4 | 0.96 | 0.14 | 5.85 | 6.62 | 2.89 | 3.55 | 0.49 | 99.9 | | | |
| 97RS-19 | 46.8 | 1.28 | 15.1 | 3.25 | 0.15 | 8.48 | 10.6 | 2.57 | 2.93 | 0.62 | 100 | 0.70851 | -15.7 | |
| 97RS-21 | 50.8 | 1.19 | 17.8 | 2.77 | 0.14 | 3.77 | 6.75 | 4.60 | 4.03 | 1.16 | 100 | 0.70819 | -12.9 | |
| 97RS-24 | 55.0 | 0.98 | 17.6 | 0.90 | 0.10 | 3.49 | 6.49 | 3.48 | 4.34 | 0.59 | 100 | | | |
| 97RS-26 | 48.6 | 1.16 | 16.4 | 1.46 | 0.13 | 7.16 | 9.47 | 1.95 | 3.23 | 0.54 | 100 | 0.70794 | -11.6 | |
| 97RS-27 | 44.7 | 1.28 | 13.4 | 2.05 | 0.18 | 10.5 | 11.9 | 2.17 | 2.16 | 0.90 | 100 | 0.70837 | -16.1 | |
| 97RY-2 | 53.0 | 0.89 | 16.6 | 2.03 | 0.12 | 5.89 | 8.44 | 2.27 | 3.40 | 0.40 | 100 | 0.70889 | -17.2 | |
| 97RY-4 | 50.3 | 0.97 | 14.8 | 2.37 | 0.14 | 7.02 | 8.05 | 2.57 | 2.63 | 0.47 | 98.5 | 0.70888 | -14.9 | |
| 97RY-6 | 53.4 | 0.85 | 15.1 | 0.91 | 0.16 | 7.68 | 7.01 | 2.58 | 2.87 | 0.38 | 99.6 | 0.70934 | -16.7 | |
| 97RY-13 | 52.9 | 0.99 | 15.3 | 1.07 | 0.14 | 7.73 | 6.62 | 2.72 | 3.25 | 0.45 | 100 | 0.70910 | -15.5 | |
| 97RY-24 | 51.4 | 0.88 | 15.0 | 2.29 | 0.12 | 7.40 | 7.79 | 2.39 | 2.31 | 0.32 | 99.9 | 0.70864 | -16.7 | |
| 97RY-25 | 49.9 | 0.99 | 14.0 | 2.33 | 0.13 | 9.14 | 8.44 | 2.07 | 2.13 | 0.29 | 100 | | | |
| 17XS01 | 47.6 | 0.75 | 13.4 | 7.63 | 0.11 | 9.32 | 7.60 | 2.14 | 1.97 | 0.24 | 100 | 0.70928 | -15.1 | [19] |
| 17XS02 | 45.7 | 0.83 | 12.9 | 8.07 | 0.13 | 10.9 | 8.10 | 2.03 | 2.27 | 0.35 | 100 | 0.70930 | -15.5 | |
| 17XS03 | 50.0 | 0.81 | 14.2 | 7.55 | 0.12 | 7.92 | 8.06 | 2.38 | 2.51 | 0.34 | 100 | | | |
| 17XS05 | 50.6 | 0.78 | 13.9 | 7.70 | 0.12 | 8.84 | 8.14 | 2.55 | 2.21 | 0.29 | 99.2 | 0.71026 | -14.8 | |
| 17XS06 | 45.2 | 0.71 | 12.4 | 7.63 | 0.12 | 10.3 | 7.77 | 1.93 | 2.17 | 0.23 | 99.7 | 0.70909 | -12.9 | |
| 17XS08 | 49.3 | 0.81 | 14.1 | 6.88 | 0.12 | 6.92 | 7.39 | 2.66 | 2.27 | 0.35 | 99.2 | 0.71020 | -15.8 | |

Continued

| | | | | | | | | | | | | | | |
|---|---|---|---|---|---|---|---|---|---|---|---|---|---|---|
| 17XS09 | 50.1 | 0.78 | 14.1 | 6.68 | 0.12 | 6.48 | 7.80 | 2.61 | 2.32 | 0.33 | 99.9 | | | |
| 17XS11 | 50.5 | 0.79 | 14.2 | 6.63 | 0.13 | 6.39 | 8.14 | 2.53 | 1.77 | 0.34 | 99.4 | | | |
| 17XS12 | 49.5 | 0.78 | 14.1 | 6.46 | 0.13 | 6.22 | 7.09 | 2.62 | 2.58 | 0.34 | 99.6 | 0.71045 | -16.2 | |
| 17XS14 | 48.3 | 0.83 | 13.6 | 7.93 | 0.12 | 8.83 | 8.68 | 2.40 | 1.81 | 0.30 | 98.9 | | | |
| 17XS16 | 49.5 | 0.60 | 13.4 | 6.55 | 0.09 | 7.50 | 6.42 | 2.30 | 2.37 | 0.19 | 99.5 | | | |
| 17XS18 | 47.1 | 0.71 | 13.4 | 7.72 | 0.12 | 9.65 | 7.96 | 1.94 | 2.30 | 0.25 | 99.4 | | | |
| 17XS19 | 45.0 | 0.67 | 13.0 | 5.71 | 0.12 | 5.84 | 9.25 | 0.87 | 3.12 | 0.21 | 99.1 | 0.71097 | -15.7 | |
| 17XS23 | 50.8 | 0.65 | 13.8 | 6.81 | 0.10 | 8.02 | 6.75 | 3.27 | 1.86 | 0.20 | 99.1 | 0.70983 | -16.5 | |
| 17XS25 | 49.4 | 0.63 | 14.1 | 6.79 | 0.11 | 7.20 | 7.60 | 2.41 | 2.09 | 0.20 | 99.5 | 0.70977 | -16.8 | |
| 17XS26 | 48.5 | 0.89 | 14.3 | 8.17 | 0.13 | 9.22 | 8.42 | 2.48 | 2.10 | 0.38 | 99.2 | 0.70903 | -15.9 | |
| 17XS27 | 50.9 | 0.80 | 14.3 | 7.52 | 0.12 | 9.25 | 7.34 | 2.91 | 2.27 | 0.26 | 99.1 | 0.70896 | -15.2 | |
| XZ4 | 47.4 | 1.67 | 17.8 | | 0.17 | 5.30 | 7.20 | 4.25 | 4.15 | 1.28 | 100 | 0.70523 | -10.5 | [38] |
| WA-26 | 56.4 | 1.26 | 16.1 | | 0.15 | 4.18 | 5.91 | 5.63 | 2.50 | 0.45 | 101 | 0.70683 | -15.2 | |
| YM-2 | 46.6 | 1.75 | 17.4 | | 0.17 | 6.20 | 8.37 | 3.76 | 3.97 | 1.06 | 100 | 0.70520 | -8.3 | |
| WA-37 | 56.1 | 1.22 | 15.4 | | 0.10 | 5.37 | 5.29 | 4.28 | 2.91 | 0.53 | 100 | 0.70554 | -8.6 | |
| DH-26 | 58.6 | 0.88 | 16.8 | | 0.11 | 2.99 | 5.63 | 3.74 | 3.02 | 0.29 | 100 | 0.70623 | -13.4 | |
| DH-29 | 63.6 | 0.68 | 16.0 | | 0.08 | 1.65 | 3.45 | 4.13 | 3.47 | 0.20 | 99.6 | 0.70659 | -14.9 | |
| 13HJS-1-1 | 42.5 | 0.91 | 12.3 | 7.63 | 0.12 | 8.04 | 10.4 | 2.03 | 5.74 | 1.25 | 90.9 | | | [39] |
| 13HJS-1-2 | 46.2 | 1.00 | 12.8 | 7.85 | 0.13 | 9.46 | 9.7 | 2.47 | 4.98 | 1.37 | 95.9 | | | |
| 13HJS-1-3 | 46.0 | 1.01 | 12.8 | 8.06 | 0.15 | 9.44 | 9.61 | 2.59 | 5.14 | 1.38 | 96.1 | | | |
| 13HJS-1-4 | 45.4 | 0.99 | 12.7 | 7.85 | 0.16 | 9.23 | 10.4 | 2.32 | 5.08 | 1.40 | 95.5 | | | |
| 11-JS-lam | 47.3 | 0.87 | 16.6 | 6.57 | 0.13 | 9.54 | 9.53 | 1.99 | 4.99 | 1.40 | 98.9 | | | |
| CE-9 | 48.6 | 1.71 | 14.5 | 12.9 | 0.19 | 6.37 | 7.69 | 2.19 | 0.79 | 0.22 | 99.6 | | | [40] |
| CE-10 | 46.7 | 1.64 | 16.0 | 12.8 | 0.16 | 7.27 | 7.27 | 2.01 | 0.41 | 0.20 | 100 | | | |
| CE-16 | 34.3 | 0.69 | 17.7 | 22.2 | 0.15 | 11.8 | 0.52 | 0.08 | 10.3 | 0.11 | 99.2 | | | |
| WY-5C | 53.9 | 3.14 | 12.6 | 15.1 | 0.23 | 2.62 | 6.53 | 1.90 | 2.38 | 0.56 | 100 | | | |

Continued

| | | | | | | | | | | | | | | |
|---|---|---|---|---|---|---|---|---|---|---|---|---|---|---|
| *Cenozoic basalts in Shandong* | | | | | | | | | | | | | | |
| QX01 | 41.5 | | | 15.3 | | | | | | | | 0.70378 | 0.4 | [41] |
| QX03 | 41.3 | | | 15.3 | | | | | | | | 0.70381 | 0.4 | |
| QX08 | 41.5 | | | 15.4 | | | | | | | | 0.70370 | 0.5 | |
| QX07 | 42.2 | | | 15.4 | | | | | | | | 0.70374 | 0.5 | |
| PL01 | 47.1 | | | 12.9 | | | | | | | | 0.70423 | 0.3 | |
| PL02 | 46.8 | | | 12.9 | | | | | | | | 0.70410 | 0.3 | |
| PL05 | 44.7 | | | 13.5 | | | | | | | | 0.70396 | 0.4 | |
| PL06 | 46.2 | | | 14.0 | | | | | | | | 0.70418 | 0.4 | |
| PL08 | 49.1 | | | 12.5 | | | | | | | | 0.70434 | 0.3 | |
| PL17 | 40.2 | | | 16.3 | | | | | | | | 0.70351 | 0.5 | |
| PL18 | 40.4 | | | 16.3 | | | | | | | | 0.70350 | 0.5 | |
| *Early Cretaceous volcanic rocks in eastern NCC* | | | | | | | | | | | | | | |
| SFX02 | 48.8 | 1.09 | 12.1 | 8.94 | 0.12 | 12.7 | 8.82 | 2.37 | 2.29 | 0.68 | 99.4 | | | [42] |
| SFX13 | 48.6 | 1.10 | 11.8 | 8.85 | 0.12 | 13.6 | 8.78 | 1.93 | 2.62 | 0.66 | 99.5 | 0.70990 | -13.1 | |
| SFX19 | 48.7 | 1.10 | 12.0 | 8.86 | 0.12 | 13.4 | 8.79 | 2.23 | 2.62 | 0.64 | 99.9 | 0.70986 | -13.1 | |
| SFX27 | 49.2 | 1.07 | 11.9 | 8.75 | 0.12 | 13.0 | 8.74 | 2.18 | 2.72 | 0.64 | 99.3 | 0.70984 | -13.2 | |
| SFX28 | 49.5 | 1.07 | 11.9 | 8.74 | 0.12 | 13.1 | 8.76 | 2.19 | 2.53 | 0.65 | 99.5 | 0.70981 | -13.3 | |
| SFX30 | 48.9 | 1.08 | 11.7 | 8.92 | 0.12 | 13.6 | 8.84 | 2.43 | 2.08 | 0.65 | 99.8 | 0.70977 | -13.4 | |
| SFX-49 | 48.5 | 1.13 | 11.7 | 8.95 | 0.12 | 13.6 | 8.87 | 2.05 | 2.24 | 0.68 | 99.8 | 0.70983 | -13.2 | |
| 07FC-1 | 49.6 | 1.15 | 14.4 | 8.72 | 0.13 | 8.88 | 8.52 | 3.36 | 1.81 | 0.95 | 100 | 0.70957 | -14.7 | [43] |
| 07FC-2 | 49.4 | 1.13 | 14.4 | 8.80 | 0.13 | 8.82 | 8.57 | 3.31 | 1.81 | 0.89 | 100 | 0.70956 | -14.8 | |
| 07FC-7 | 59.0 | 0.72 | 15.2 | 5.52 | 0.08 | 4.71 | 6.01 | 3.61 | 2.17 | 0.29 | 100 | 0.70968 | -15.0 | |
| SHT-16 | 49.9 | 0.86 | 12.6 | 8.40 | 0.12 | 12.3 | 7.30 | 2.69 | 2.30 | 0.67 | 99.5 | 0.70627 | -2.1 | [42] |
| SHT-19 | 49.8 | 0.84 | 13.0 | 8.53 | 0.11 | 11.0 | 7.83 | 2.52 | 2.44 | 0.80 | 99.6 | 0.70623 | -2.4 | |
| SHT-21 | 49.4 | 0.84 | 12.7 | 8.48 | 0.12 | 11.1 | 8.08 | 2.48 | 2.35 | 0.77 | 99.5 | 0.70624 | -1.7 | |
| SHT-24 | 50.2 | 0.82 | 12.0 | 8.31 | 0.13 | 11.9 | 7.18 | 2.68 | 3.12 | 0.73 | 99.7 | | | |

Continued

| | | | | | | | | | | | | | | |
|---|---|---|---|---|---|---|---|---|---|---|---|---|---|---|
| SHT-28 | 50.2 | 0.83 | 12.8 | 8.53 | 0.11 | 10.9 | 8.14 | 2.58 | 2.39 | 0.77 | 99.5 | 0.70622 | -1.6 | |
| SHT-31 | 50.5 | 0.87 | 12.9 | 8.36 | 0.12 | 11.5 | 7.32 | 3.01 | 2.35 | 0.67 | 99.7 | 0.70616 | -2.2 | |
| HBJ4-1 | 54.8 | 0.86 | 15.2 | 7.88 | 0.10 | 6.45 | 6.73 | 3.79 | 1.65 | 0.34 | 99.9 | 0.70624 | -10.6 | [44] |
| HBJ4-2 | 53.2 | 0.90 | 15.7 | 8.11 | 0.11 | 6.23 | 7.16 | 4.18 | 1.92 | 0.36 | 100 | 0.70629 | -12.0 | |
| HBJ4-3 | 52.8 | 0.89 | 15.6 | 8.33 | 0.09 | 7.03 | 7.17 | 4.13 | 1.91 | 0.36 | 101 | 0.70614 | -9.7 | |
| SHT-14 | 55.8 | 1.10 | 15.5 | 7.90 | 0.11 | 5.21 | 5.92 | 4.06 | 2.40 | 0.55 | 101 | 0.70587 | -13.4 | |
| SHT-3 | 56.7 | 0.74 | 15.0 | 6.26 | 0.09 | 5.92 | 5.36 | 4.26 | 2.66 | 0.46 | 100 | 0.70664 | -11.9 | |
| B046-1 | 50.8 | 1.30 | 14.4 | 4.86 | 0.11 | 7.42 | 7.21 | 3.57 | 2.08 | 0.69 | 101 | | | [45] |
| B065-2 | 56.5 | 1.74 | 16.2 | 4.77 | 0.11 | 2.55 | 5.09 | 4.57 | 3.07 | 0.82 | 99.8 | | | |
| B065-3 | 57.4 | 1.74 | 16.0 | 6.79 | 0.08 | 1.82 | 4.56 | 4.43 | 2.78 | 0.77 | 102 | | | |
| B066-1 | 57.0 | 1.75 | 16.0 | 3.75 | 0.17 | 1.85 | 5.64 | 4.64 | 3.02 | 0.83 | 101 | | | |
| B047-4 | 49.2 | 1.68 | 14.7 | 4.44 | 0.14 | 7.92 | 7.33 | 3.59 | 1.91 | 0.90 | 99.8 | | | |
| B048-1 | 18.3 | 0.67 | 14.1 | 5.03 | 0.14 | 7.57 | 7.73 | 3.20 | 1.83 | 0.81 | 100 | | | |
| B049-1 | 51.3 | 1.10 | 16.3 | 3.81 | 0.14 | 5.25 | 8.58 | 3.24 | 0.88 | 0.20 | 99.9 | | | |
| B050-3 | 50.6 | 1.39 | 15.0 | 3.92 | 0.12 | 8.33 | 6.74 | 3.39 | 2.42 | 0.91 | 99.8 | | | |
| B044-1 | 51.0 | 1.12 | 14.1 | 1.80 | 0.12 | 8.70 | 7.34 | 3.17 | 2.19 | 0.70 | 101 | | | |
| B050-2 | 50.6 | 1.37 | 14.8 | 2.85 | 0.13 | 8.25 | 6.83 | 3.50 | 2.51 | 0.93 | 100 | | | |
| B034-1 | 48.5 | 0.72 | 11.9 | 3.25 | 0.15 | 14.0 | 7.56 | 2.92 | 1.19 | 0.73 | 99.7 | 0.70702 | -7.2 | |
| B031-1 | 54.1 | 1.00 | 14.7 | 3.60 | 0.13 | 6.68 | 7.23 | 4.01 | 1.91 | 0.70 | 98.4 | 0.70613 | -11.0 | |
| B058-2 | 53.8 | 1.39 | 15.4 | 5.74 | 0.12 | 5.43 | 7.22 | 3.63 | 1.90 | 0.52 | 99.8 | 0.70620 | -11.4 | |
| B070-1 | 54.0 | 1.54 | 16.3 | 5.31 | 0.13 | 3.44 | 6.90 | 3.94 | 1.65 | 0.44 | 99.8 | 0.70569 | -9.8 | |
| 18JF13 | 51.5 | 1.97 | 17.7 | 9.60 | 0.07 | 3.16 | 3.47 | 5.09 | 3.29 | 0.59 | 99.8 | 0.70702 | −5.5 | [46] |
| 18JF14 | 52.8 | 1.96 | 17.8 | 9.50 | 0.10 | 2.85 | 4.34 | 5.55 | 2.61 | 0.58 | 100 | 0.70703 | −5.9 | |
| 18JF44 | 52.9 | 0.74 | 14.9 | 4.22 | 0.11 | 2.17 | 8.17 | 4.28 | 2.24 | 0.18 | 99.7 | 0.70881 | −0.5 | |
| 18JF45 | 55.5 | 1.42 | 14.1 | 7.87 | 0.05 | 2.77 | 3.15 | 3.31 | 2.12 | 0.25 | 97.1 | 0.70617 | −3.0 | |
| DBG-07 | 55.1 | 1.51 | 15.3 | 10.07 | 0.12 | 7.27 | 6.25 | 3.48 | 2.04 | 0.75 | 104 | 0.70591 | -11.0 | [47] |

Continued

| | | | | | | | | | | | | | | |
|---|---|---|---|---|---|---|---|---|---|---|---|---|---|---|
| DBG-08 | 51.8 | 1.47 | 14.1 | 10.01 | 0.12 | 8.49 | 5.95 | 3.39 | 2.02 | 0.71 | 100 | 0.70584 | -11.0 | |
| DBG-09 | 51.8 | 1.47 | 14.4 | 9.34 | 0.28 | 5.31 | 7.77 | 3.56 | 2.14 | 0.74 | 100 | | | |
| DBG-26 | 51.5 | 1.55 | 15.3 | 9.03 | 0.11 | 6.04 | 6.66 | 3.37 | 1.88 | 0.78 | 99.6 | | | |
| DBG-27 | 52.2 | 1.52 | 14.4 | 9.61 | 0.12 | 6.75 | 6.17 | 3.39 | 1.98 | 0.74 | 99.5 | | | |
| DBG-28 | 52.0 | 1.53 | 14.8 | 9.36 | 0.13 | 6.24 | 6.66 | 3.39 | 1.94 | 0.76 | 99.5 | | | |
| DBG-29 | 52.5 | 1.53 | 14.9 | 9.30 | 0.11 | 5.61 | 6.78 | 3.45 | 1.95 | 0.77 | 99.7 | | | |
| BNB11-01 | 56.2 | 1.90 | 13.6 | 9.76 | 0.11 | 2.99 | 6.20 | 3.67 | 2.35 | 1.10 | 99.1 | 0.70720 | -11.8 | [48] |
| BNB11-04 | 55.4 | 1.97 | 14.0 | 8.66 | 0.11 | 2.51 | 7.31 | 3.67 | 2.25 | 1.11 | 99.3 | 0.70720 | -12.2 | |
| BNB11-06 | 54.9 | 1.94 | 13.8 | 9.72 | 0.09 | 3.21 | 6.11 | 3.64 | 2.65 | 1.06 | 98.8 | 0.70731 | -11.5 | |
| 98JM-1 | 51.8 | 1.13 | 16.1 | 4.05 | 0.11 | 3.08 | 8.44 | 3.12 | 2.38 | 0.54 | 98.7 | 0.70750 | -17.0 | [49] |
| 98JM-2 | 52.4 | 1.10 | 16.5 | 3.95 | 0.10 | 3.55 | 8.11 | 3.41 | 2.35 | 0.55 | 99.0 | 0.70724 | -16.7 | |
| 98JM-3 | 54.0 | 1.13 | 16.8 | 4.07 | 0.11 | 3.68 | 7.84 | 3.23 | 2.42 | 0.54 | 99.1 | 0.70742 | -16.0 | |
| 98JM-5 | 51.7 | 1.13 | 16.2 | 2.93 | 0.12 | 4.62 | 8.57 | 2.83 | 1.78 | 0.55 | 100 | 0.70726 | -15.9 | |
| 97HX-3 | 51.8 | 1.17 | 18.2 | 4.54 | 0.10 | 4.24 | 5.48 | 3.71 | 2.80 | 0.47 | 100 | | | |
| 97HX-4 | 45.2 | 1.16 | 14.0 | 1.64 | 0.12 | 6.48 | 9.11 | 3.09 | 1.54 | 0.47 | 99.2 | | | |
| 97HX-9 | 54.6 | 1.13 | 17.1 | 1.78 | 0.11 | 5.33 | 7.53 | 2.97 | 2.87 | 0.36 | 100 | | | |
| QD-1 | 48.5 | 1.41 | 13.7 | 9.16 | 0.13 | 9.75 | 7.53 | 3.17 | 3.21 | 1.13 | 99.9 | 0.70750 | -18.1 | [50] |
| QD-2 | 48.5 | 1.43 | 13.8 | 9.24 | 0.13 | 9.67 | 7.68 | 2.87 | 2.87 | 1.11 | 99.6 | | | |
| QD-3 | 48.6 | 1.40 | 13.7 | 9.15 | 0.13 | 9.75 | 7.70 | 3.48 | 3.48 | 1.15 | 100 | 0.70725 | -17.5 | |
| QD-5 | 48.2 | 1.44 | 13.9 | 9.10 | 0.13 | 8.87 | 7.91 | 3.36 | 3.36 | 1.04 | 99.8 | 0.70794 | -17.4 | |
| QD-6 | 48.5 | 1.44 | 13.9 | 9.06 | 0.13 | 8.94 | 8.22 | 3.46 | 3.46 | 0.99 | 100 | 0.70770 | -16.5 | |
| QD-7 | 47.5 | 1.44 | 13.7 | 9.06 | 0.13 | 8.92 | 7.79 | 3.31 | 3.31 | 1.00 | 99.7 | | | |
| QD-9 | 46.2 | 1.40 | 13.4 | 8.82 | 0.13 | 8.79 | 7.39 | 3.12 | 3.12 | 0.97 | 99.9 | 0.70799 | -16.5 | |
| JZ-5 | 54.3 | 1.39 | 17.0 | 7.78 | 0.12 | 3.90 | 6.43 | 2.97 | 2.97 | 0.67 | 99.9 | | | |
| JZ-6 | 54.4 | 1.40 | 17.2 | 7.55 | 0.12 | 3.72 | 6.47 | 2.99 | 2.99 | 0.64 | 99.7 | 0.70718 | -18.0 | |
| JZ-7 | 54.3 | 1.40 | 13.1 | 7.78 | 1.09 | 3.96 | 6.41 | 2.97 | 2.97 | 0.67 | 100 | | | |
| JM-8 | 51.5 | 1.19 | 16.6 | 6.95 | 0.08 | 6.55 | 7.93 | 1.94 | 1.94 | 0.22 | 100 | | | |

Continued

| | | | | | | | | | | | | | | |
|---|---|---|---|---|---|---|---|---|---|---|---|---|---|---|
| JM-9 | 50.2 | 1.09 | 15.0 | 8.16 | 0.09 | 8.03 | 8.76 | 1.37 | 1.37 | 0.28 | 99.9 | 0.70738 | -15.3 | |
| JM-10 | 51.4 | 1.12 | 16.5 | 7.39 | 0.07 | 5.56 | 9.15 | 1.39 | 1.39 | 0.24 | 100 | | | |
| JM-11 | 49.1 | 1.18 | 16.2 | 11.1 | 0.05 | 8.89 | 1.88 | 0.65 | 0.65 | 0.14 | 100 | | | |
| JM-13 | 44.9 | 1.03 | 15.5 | 7.82 | 0.10 | 8.62 | 6.53 | 0.76 | 0.76 | 0.14 | 99.5 | | | |
| JM-14 | 51.2 | 1.01 | 15.3 | 9.38 | 0.05 | 6.30 | 4.61 | 0.78 | 0.78 | 0.11 | 100 | | | |
| JM-15 | 52.2 | 1.11 | 16.3 | 6.89 | 0.06 | 6.67 | 8.14 | 1.40 | 1.40 | 0.20 | 100 | 0.70788 | -15.8 | |
| JM-16 | 47.0 | 1.15 | 16.8 | 7.23 | 0.10 | 7.59 | 8.95 | 2.59 | 0.99 | 0.19 | 100 | 0.70785 | -18.9 | |
| JM-17 | 47.0 | 1.15 | 16.7 | 7.69 | 0.10 | 8.18 | 8.13 | 2.46 | 0.98 | 0.18 | 100 | | | |
| JM-18 | 52.6 | 1.05 | 15.6 | 8.40 | 0.07 | 8.10 | 5.99 | 2.53 | 1.00 | 0.13 | 100 | 0.70797 | -15.4 | |
| HY-2 | 51.2 | 0.96 | 14.9 | 9.54 | 0.07 | 8.36 | 5.18 | 2.59 | 2.72 | 0.30 | 100 | 0.70884 | -19.1 | |
| HY-3 | 51.7 | 1.03 | 13.8 | 7.81 | 0.09 | 6.87 | 6.51 | 2.97 | 4.03 | 0.63 | 100 | | | |
| HY-4 | 53.9 | 0.99 | 13.9 | 7.66 | 0.08 | 6.65 | 5.59 | 2.48 | 3.85 | 0.61 | 100 | 0.70929 | -19.6 | |
| HY-5 | 51.6 | 0.98 | 13.6 | 7.60 | 0.07 | 6.14 | 5.76 | 3.31 | 3.13 | 0.65 | 99.9 | 0.70949 | -19.4 | |
| HY-6 | 50.8 | 1.00 | 14.0 | 8.21 | 0.08 | 7.01 | 5.51 | 3.56 | 2.30 | 0.65 | 100 | | | |
| HY-9 | 52.0 | 1.00 | 13.6 | 7.82 | 0.09 | 5.99 | 6.18 | 3.22 | 2.75 | 0.59 | 101 | 0.70947 | -19.5 | |
| HY-10 | 53.3 | 1.17 | 16.2 | 7.27 | 0.08 | 4.14 | 4.02 | 3.99 | 4.62 | 0.52 | 99.6 | 0.70791 | -19.1 | |
| LJZ-2 | 59.0 | 1.01 | 18.5 | 5.74 | 0.04 | 0.83 | 2.66 | 5.31 | 3.21 | 0.76 | | 0.70632 | -17.9 | |
| LJZ-5 | 49.2 | 1.44 | 16.7 | 9.68 | 0.17 | 5.56 | 8.68 | 2.90 | 2.55 | 0.55 | | 0.70596 | -10.3 | |
| LJZ-7 | 51.8 | 1.44 | 18.2 | 7.87 | 0.08 | 3.57 | 5.34 | 4.06 | 3.79 | 0.70 | | 0.70587 | -12.3 | |
| LJZ-15 | 58.2 | 0.90 | 16.9 | 6.54 | 0.05 | 2.44 | 3.95 | 5.42 | 2.99 | 0.71 | | 0.70579 | -18.9 | |
| S7123 | 52.2 | 2.01 | 15.7 | 8.70 | 0.14 | 5.11 | 7.64 | 3.85 | 2.88 | 0.88 | 104 | | | [51] |
| S7125 | 54.4 | 1.19 | 15.4 | 6.45 | 0.76 | 1.64 | 10.7 | 4.33 | 3.74 | 0.75 | 107 | | | |
| N07-1 | 54.6 | 1.29 | 16.9 | 8.43 | 0.16 | 2.85 | 5.53 | 4.45 | 3.98 | 0.83 | 102 | | | |
| N08-1 | 55.4 | 1.18 | 15.7 | 7.45 | 0.18 | 2.72 | 7.75 | 4.19 | 3.97 | 0.70 | 104 | | | |
| WH1007 | 51.9 | 1.75 | 15.6 | 10.4 | 0.16 | 6.10 | 6.39 | 3.68 | 2.25 | 0.61 | 99.8 | | | |
| WH1009 | 53.5 | 1.35 | 15.8 | 9.30 | 0.18 | 5.56 | 3.74 | 4.37 | 4.29 | 0.90 | 99.8 | | | |
| WH1008 | 54.7 | 1.20 | 16.1 | 8.23 | 0.10 | 4.78 | 5.78 | 5.02 | 2.52 | 0.75 | 99.8 | | | |

Continued

| | | | | | | | | | | | |
|---|---|---|---|---|---|---|---|---|---|---|---|
| S7056 | 51.3 | 2.73 | 16.7 | 11.8 | 0.19 | 2.92 | 6.27 | 4.14 | 2.14 | 0.52 | 99.8 |
| S7058 | 48.5 | 2.26 | 16.1 | 13.1 | 0.18 | 4.76 | 8.64 | 3.35 | 1.30 | 0.38 | 99.9 |
| S7065 | 50.1 | 2.14 | 14.6 | 11.9 | 0.17 | 6.42 | 8.74 | 3.14 | 1.18 | 0.35 | 99.9 |
| S6078 | 46.4 | 2.93 | 13.6 | 17.2 | 0.20 | 5.16 | 8.22 | 2.92 | 1.07 | 0.41 | 106 |
| S4151 | 51.4 | 2.31 | 16.3 | 13.1 | 0.14 | 2.64 | 4.93 | 4.38 | 2.76 | 0.55 | 103 |
| S6084 | 49.0 | 1.68 | 14.5 | 10.7 | 0.15 | 5.74 | 7.52 | 4.09 | 4.11 | 1.33 | 100 |
| S6086 | 51.4 | 2.00 | 14.8 | 10.4 | 0.13 | 5.32 | 7.80 | 3.47 | 2.38 | 1.13 | 100 |
| S6076 | 52.5 | 1.81 | 12.3 | 11.1 | 0.13 | 7.04 | 7.46 | 3.53 | 2.46 | 0.50 | 103 |

**Table S3.** Mg, Sr and Nd isotope compositions of the studied lamprophyres.

| Sample No. | $\delta^{25}$Mg (‰)[a] | 2SD | $\delta^{26}$Mg (‰)[a] | 2SD | $^{87}Sr/^{86}Sr$ | $^{87}Sr/^{86}Sr_{(i)}$[b] | $^{143}Nd/^{144}Nd$ | $^{143}Nd/^{144}Nd_{(t)}$ | $\varepsilon_{Nd}(t)$[c] |
|---|---|---|---|---|---|---|---|---|---|
| *High-MgO lamprophyres* | | | | | | | | | |
| HY-11 | -0.21 | 0.03 | -0.47 | 0.05 | 0.70844 | 0.70832 | 0.51162 | 0.51153 | -18.4 |
| QX-01 | -0.18 | 0.04 | -0.42 | 0.04 | 0.71072 | 0.71057 | 0.51172 | 0.51164 | -16.4 |
| LL06 | -0.20 | 0.02 | -0.37 | 0.02 | 0.70999 | 0.70987 | 0.51165 | 0.51156 | -17.8 |
| PD03 | -0.31 | 0.02 | -0.59 | 0.01 | 0.70983 | 0.70905 | 0.51171 | 0.51163 | -16.6 |
| HY04 | -0.18 | 0.02 | -0.38 | 0.03 | 0.70846 | 0.70822 | 0.51192 | 0.51184 | -12.4 |
| HY-12 | -0.15 | 0.01 | -0.35 | 0.03 | 0.70864 | 0.70848 | 0.51194 | 0.51185 | -12.1 |
| QX-04 | -0.15 | 0.03 | -0.39 | 0.01 | 0.70942 | 0.70917 | 0.51173 | 0.51165 | -16.1 |
| *Low-MgO lamprophyres* | | | | | | | | | |
| LZ04 | -0.17 | 0.01 | -0.31 | 0.02 | 0.70762 | 0.70730 | 0.51173 | 0.51165 | -16.1 |
| PD08 | -0.13 | 0.04 | -0.27 | 0.04 | 0.70748 | 0.70602 | 0.51188 | 0.51179 | -13.3 |
| LL01 | -0.15 | 0.02 | -0.28 | 0.01 | 0.70961 | 0.70941 | 0.51166 | 0.51158 | -17.6 |
| HY06 | -0.16 | 0.02 | -0.32 | 0.03 | 0.70840 | 0.70805 | 0.51185 | 0.51177 | -13.8 |
| HY03 | -0.14 | 0.01 | -0.29 | 0.03 | 0.70871 | 0.70836 | 0.51185 | 0.51177 | -13.7 |
| LZ02 | -0.13 | 0.04 | -0.28 | 0.02 | 0.70919 | 0.70876 | 0.51199 | 0.51191 | -11.1 |
| QX-03 | -0.13 | 0.02 | -0.32 | 0.01 | 0.70962 | 0.70945 | 0.51163 | 0.51155 | -18.0 |
| LL04 | -0.13 | 0.03 | -0.24 | 0.03 | 0.71008 | 0.70743 | 0.51162 | 0.51154 | -18.3 |
| PD04 | -0.16 | 0.01 | -0.31 | 0.01 | 0.70759 | 0.70727 | 0.51189 | 0.51182 | -12.9 |
| BHVO-2 | -0.13 | 0.03 | -0.24 | 0.04 | 0.703468 | | 0.51295 | | |
| BCR-2 | -0.11 | 0.02 | -0.17 | 0.04 | 0.705119 | | 0.51262 | | |

[a] $\delta^{X}Mg$ (‰) = $[(^{X}Mg/^{24}Mg)_{sample}/(^{X}Mg_{sample}/^{24}Mg)_{DSM3} - 1] \times 1000$ (X=25 or 26)

[b] $^{87}Sr/^{86}Sr_{(i)} = {}^{87}Sr/^{86}Sr - {}^{87}Rb/^{86}Sr \times [EXP(125\times10^{6}\times1.42\times10^{-11})-1]$ ($i$=125 Ma)

[c] $\varepsilon_{Nd}(t) = [(^{143}Nd/^{144}Nd_{(t)})_{sample}/(^{143}Nd/^{144}Nd_{(t)})_{CHUR} - 1] \times 10000$ ($t$=125 Ma)

**Table S4.** Parameters of end members for mixing modelling in this study.

| End-Member | MgO (wt %) | $\delta^{26}$Mg (‰) | Sr (ppm) | $^{87}Sr/^{86}Sr$ |
|---|---|---|---|---|
| Depleted Mantle[a] | 38 | -0.25 | 7.7 | 0.7025 |
| Dolomite[b] | 22 | -2.0 | 1311 | 0.7099 |
| Sediment[c] | 2.75 | -0.05 | 302 | 0.7124 |

[a]The average MgO, Sr and $^{87}Sr/^{86}Sr$ are from [52], $\delta^{26}$Mg are from [53].

[b]The MgO and $\delta^{26}$Mg of dolomite are taken from [22], Sr and $^{87}Sr/^{86}Sr$ are from [54]

[c]The MgO, Sr contents and $^{87}Sr/^{86}Sr$ ratios are from [55], and the average $\delta^{26}$Mg of carbonate-free sediments is from [56].

**Table S5.** Chemical compositions of Cpx in Paleozoic kimberlites and Mesozoic rocks.

| Sample No. | Mg# | Ca/Al | Ti/Eu | $(La/Yb)_N$ | reference |
|---|---|---|---|---|---|
| MORB | 90.8 | 3.35 | 4441 | 0.63 | [52] |
| *CPX in Paleozoic kimberlites* | | | | | |
| MY0211 | 94.6 | 27.0 | | | [57] |
| MY0278 | 92.8 | 16.5 | | | |
| MY0211 | | | 375 | 18.3 | |
| MY0278 | | | 461 | 13.8 | |
| *CPX in Mesozoic mafic rocks (>110 Ma)* | | | | | |
| Y97-0 | 93.0 | 8.01 | 290 | 59.8 | [58] |
| Y97-1 | 93.0 | 9.88 | 545 | 24.4 | |
| Y97-2 | 93.0 | 8.25 | 362 | 30.9 | |
| Y97-4 | 93.0 | 7.05 | 416 | 27.0 | |
| Y97-5 | 92.0 | 4.37 | 527 | 8.71 | |
| FS7-10 | 95.0 | 17.1 | | | [59] |
| FS7-10 | 95.0 | 13.7 | | | |
| FS6-18 | 94.0 | 23.5 | | | |
| FS6-55 | 94.0 | 5.49 | | | |
| FS7-1 | 93.0 | 5.60 | | | |
| FS7-9 | 94.0 | 13.4 | | | |
| T7-21-2.2 | 95.0 | 23.7 | | | [60] |
| T7-21-4.4 | 93.0 | 16.1 | | | |
| LW8-44-1.8 | 92.0 | 21.1 | | | |
| LW8-44-2.3 | 91.0 | 19.4 | | | |
| LW8-47-1.5 | 95.0 | 11.9 | | | |
| LW8-43-2.1 | 91.0 | 17.9 | | | |
| LW8-43-3.1 | 90.0 | 17.4 | | | |
| LW8-43-2.2 | 92.0 | 21.3 | | | |
| LW8-41-3.2 | 94.0 | 109 | | | |
| LW8-45-1.1 | 92.0 | 24.2 | | | |
| LW8-49-2.3 | 92.0 | 33.5 | 2471 | 4.13 | [61] |
| LW8-49-2.4 | 92.0 | 34.7 | 2211 | 5.56 | |
| LW9-23-1 | 93.2 | 38.4 | 3000 | 6.06 | |
| LW9-23-2 | 92.9 | 39.1 | 1875 | 4.90 | |
| LW9-23-3 | 92.9 | 28.7 | 5143 | 5.28 | |
| LW9-23-4 | 92.7 | 26.2 | 5143 | 4.45 | |
| LW9-23-5 | 92.5 | 44.9 | 2250 | 5.83 | |
| LW9-23-6 | 91.2 | 25.8 | 3000 | 4.81 | |
| LW9-23-7 | 93.1 | 36.3 | 3818 | 5.84 | |
| LW9-23-8 | 93.3 | 37.3 | 4000 | 6.46 | |
| LW9-23-9 | 92.3 | 33.6 | 2250 | 5.44 | |
| LW9-23-10 | 92.6 | 33.7 | 1333 | 4.72 | |
| LW9-23-11 | 92.6 | 37.9 | 1412 | 4.66 | |
| LW9-23-12 | 93.2 | 42.1 | | | |
| LW8-49-1 | 93.6 | 32.6 | | | |

Continued

| | | | | | |
|---|---|---|---|---|---|
| LW8-49-2 | 94.1 | 33.3 | | | |
| LW8-49-3 | 93.0 | 32.5 | | | |
| LW8-49-4 | 93.4 | 32.6 | | | |
| LW8-49-5 | 93.1 | 34.3 | | | |
| LW8-49-6 | 93.5 | 29.9 | | | |
| LW8-49-7 | 93.5 | 33.2 | | | |
| LW8-49-8 | 93.1 | 35.6 | | | |
| LW8-49-9 | 93.0 | 36.1 | | | |
| LW8-49-10 | 92.7 | 32.5 | | | |
| LW8-49-11 | 93.2 | 36.2 | | | |
| LW8-49-12 | 93.1 | 30.4 | | | |
| FC-3-① | 88.4 | 10.2 | 1330 | 6.54 | [62] |
| FC-3-② | 88.4 | 10.4 | 1225 | 7.79 | |
| FC-67-① | 84.6 | 10.2 | 1303 | 11.9 | |
| FC-67-② | 84.3 | 7.94 | 1541 | 12.1 | |
| FC-67-③ | 86.7 | 11.2 | 1737 | 10.8 | |
| FC-67-④ | 84.8 | 8.81 | 1545 | 12.3 | |
| FC-11-① | 85.0 | 11.1 | 1351 | 8.58 | |
| FC-11-② | 84.6 | 8.71 | 1640 | 7.95 | |
| FC-11-③-1 | 84.5 | 8.79 | 1470 | 6.78 | |
| FC-11-③-2 | 84.7 | 10.1 | 1387 | 9.76 | |
| FC-11-④ | 85.4 | 11.0 | 1362 | 9.26 | |
| FC-11-⑤ | 85.8 | 11.2 | 1401 | 11.3 | |
| FC-11-1 | 84.7 | 9.68 | | | |
| FC-11-2 | 85.5 | 10.4 | | | |
| FC-3-1 | 88.1 | 9.43 | | | |
| FC-3-2 | 88.3 | 8.55 | | | |
| FC-67-1 | 84.2 | 10.6 | | | |
| FC-67-2 | 85.0 | 20.0 | | | |
| FC-67-3 | 83.8 | 7.80 | | | |
| *FC-09-5-1 | 83.4 | 7.23 | | | |
| *FC-09-5-2 | 83.4 | 7.35 | | | |
| FC09-5-① | 83.8 | 7.27 | 1276 | 5.93 | |
| FC09-5-② | 83.7 | 7.11 | 1306 | 6.74 | |
| FC09-5-③ | 84.1 | 7.38 | 1218 | 6.38 | |
| *FC-30-1 | 86.5 | 9.20 | | | |
| *FC-30-2 | 86.0 | 10.4 | | | |
| *FC-30-3 | 86.2 | 11.3 | | | |
| FC-30-① | 86.4 | 10.5 | 1422 | 9.41 | |
| FC-30-② | 86.7 | 11.0 | 1341 | 9.06 | |
| FC-30-③ | 84.7 | 9.68 | 1304 | 11.5 | |

Continued

| | | | | | |
|---|---|---|---|---|---|
| *FC-31-1 | 78.6 | 6.78 | | | |
| *FC-31-2 | 88.4 | 12.9 | | | |
| *FC-31-3 | 88.2 | 13.4 | | | |
| FC-31-① | 89.0 | 13.3 | 1074 | 13.0 | |
| FC-31-② | 88.3 | 13.0 | 941 | 13.2 | |
| FX2-57-6 | 88.3 | 11.6 | 1661 | 6.30 | |
| FX2-57-9 | 88.4 | 13.3 | 1500 | 5.45 | |
| *11FX03-1 | 89.1 | 6.27 | | | |
| *11FX03-2 | 89.0 | 6.15 | | | |
| 11FX03-1 | 88.7 | 5.99 | 1175 | 6.74 | |
| 11FX03-2 | 88.6 | 6.04 | 1225 | 7.68 | |
| 11FX03-3 | 88.6 | 6.05 | 1261 | 5.90 | |
| 11FX03-4 | 88.5 | 5.99 | 1200 | 6.48 | |
| 11FX03-5 | 88.5 | 5.99 | 1209 | 5.47 | |
| *11FX12-1 | 86.7 | 4.40 | | | |
| *11FX12-2 | 86.3 | 4.24 | | | |
| *11FX12-3 | 87.1 | 4.39 | | | |
| *11FX12-4 | 86.9 | 4.56 | | | |
| 11FX12-1 | 86.2 | 4.35 | 1162 | 7.42 | |
| 11FX12-2 | 86.2 | 4.32 | 1298 | 6.19 | |
| *11FX19-1 | 89.2 | 10.9 | | | |
| *11FX19-2 | 89.5 | 11.1 | | | |
| *11FX19-3 | 78.0 | 8.01 | | | |
| FX-75 (n= 70) | 90.4 | 11.5 | 1562 | 8.18 | [63] |
| FX69-1-1 | 87.9 | 6.70 | 1721 | 7.05 | |
| FX69-1-3.2 | 87.6 | 6.56 | 1617 | 9.19 | |
| FX69-1-4.1 | 87.8 | 6.21 | 1846 | 6.97 | |
| FX69-1-4.2 | 87.6 | 6.57 | 1932 | 6.48 | |
| FX4-2-3.1 | 89.1 | 8.61 | 1266 | 6.51 | |
| FX4-2-3.2 | 89.3 | 8.88 | 1429 | 6.27 | |
| *CPX in Mesozoic alkali rocks (<110 Ma)* | | | | | |
| JG17-01 | 92.0 | 7.55 | 1230 | 4.32 | [64] |
| JG17-05 | 92.6 | 9.90 | 1002 | 7.01 | |
| JG17-02 | 91.0 | 5.77 | 2335 | 2.16 | |
| JG17-03 | 89.5 | 4.88 | 2206 | 2.40 | |
| JG17-04 | 89.2 | 4.75 | 2715 | 1.27 | |
| JG17-09 | 90.9 | 5.35 | 2076 | 2.45 | |
| JG19-01 | 89.0 | 3.05 | 5857 | 0.83 | |
| JG19-02 | 89.5 | 4.87 | 1766 | 2.31 | |
| JG19-03 | 89.4 | 4.66 | 2482 | 2.03 | |
| JG19-05 | 90.3 | 5.76 | 1307 | 2.73 | |
| JG19-06 | 84.4 | 5.19 | 5630 | 1.92 | |
| JG19-07 | 90.3 | 6.85 | 1442 | 2.41 | |
| JG6 | 90.6 | 6.61 | 2870 | 2.33 | [65] |

Continued

| | | | | | |
|---|---|---|---|---|---|
| JG17 | 89.4 | 32.5 | 1817 | 4.46 | |
| JG18 | 90.7 | 6.24 | 2526 | 2.02 | |
| JG19 | 91.0 | 9.43 | 1841 | 1.50 | |
| JG19 | 90.4 | 5.08 | | | |
| JG20 | 90.4 | 6.83 | 1909 | 1.21 | |
| JG26 | 91.2 | 6.95 | 1291 | 4.11 | |
| JG27 | 91.4 | 6.17 | 3857 | 1.05 | |
| JG28 | 92.4 | 12.1 | 3429 | 0.95 | |
| JG28 | 90.4 | 6.12 | | | |
| JG29 | 91.1 | 5.72 | 2459 | 1.48 | |
| JG10 | 88.1 | 8.13 | 2339 | 3.06 | |
| JG12 | 89.9 | 6.31 | | | |
| JG13-1 | 86.2 | 7.67 | 723 | 6.05 | |
| JG13-2 | 87.2 | 7.18 | 3055 | 5.86 | |
| F1a | 90.6 | 3.12 | 3481 | 0.06 | |
| F20ma | 90.4 | 3.92 | 4310 | 0.41 | |
| JL1-3 | 89.5 | 4.52 | 5077 | 0.96 | [66] |
| JL1-12 | 89.7 | 4.64 | 5829 | 0.76 | |
| JL1-12 | 89.8 | 4.62 | 6000 | 0.63 | |
| JL1-13 | 90.2 | 9.35 | 968 | 37.7 | |
| JL1-13 | 90.1 | 9.48 | 818 | 12.8 | |
| JL7-2 | 90.8 | 4.69 | 4246 | 0.07 | |
| PSK03-24 | 92.9 | 8.33 | | | [67] |
| PSK03-32 | 92.9 | 8.29 | | | |
| PSK03-33 | 91.3 | 4.59 | | | |
| PSK03-43 | 91.7 | 6.15 | 3088 | 15.4 | |
| PSK03-44 | 92.1 | 11.9 | | | |
| PSK03-45 | 92.6 | 7.91 | | | |
| PSK03-48 | 91.6 | 6.16 | 3529 | 15.2 | |
| PSK03-49 | 92.2 | 8.07 | | | |
| PSK03-410 | 92.1 | 11.3 | 462 | 37.6 | |
| PSK03-414 | 93.0 | 10.3 | | | |
| PSK03-1211 | 92.0 | 5.68 | | | |
| PSK04-23 | 91.2 | 4.90 | | | |
| PSK04-168 | 92.3 | 6.62 | | | |
| PSK04-213 | 90.9 | 5.65 | | | |
| PSK04-216 | 91.1 | 5.79 | | | |
| PSK04-217 | 90.8 | 5.68 | | | |
| PSK04-225 | 91.6 | 7.33 | | | |
| PSK03-1212 | 90.8 | 4.95 | 4839 | 0.29 | |
| PSK03-124 | 91.1 | 5.05 | | | |
| PSK04-24 | 90.3 | 4.65 | | | |
| PSK04-31 | 90.7 | 5.33 | | | |
| PSK04-137 | 90.7 | 4.79 | | | |
| 06DXZ-02 | 91.2 | 7.97 | 5000 | 1.09 | [68] |

Continued

| | | | | | |
|---|---|---|---|---|---|
| 06DXZ-03 | 90.1 | 5.09 | 4800 | 0.26 | |
| 06DXZ-07 | 91.3 | 7.93 | 2634 | 2.89 | |
| 06DXZ-16 | 91.1 | 5.45 | 2067 | 8.61 | |
| 06DXZ-17 | 89.8 | 4.16 | 4200 | 0.56 | |
| 06DXZ-28 | 90.9 | 4.41 | 4925 | 13.2 | |
| 06DXZ-15 | 89.7 | 6.07 | 469 | 4.96 | |
| 06DXZ-21 | 88.6 | 6.78 | 940 | 4.41 | |
| 03DXZ-01 | 88.4 | 10.8 | 1403 | 4.99 | |
| 06DXZ-12 | 86.5 | 3.10 | 11333 | 2.69 | |
| 06DXZ-05 | 86.1 | 3.65 | 1645 | 2.72 | |
| 03DXZ-03 | 86.7 | 5.13 | 16071 | 3.63 | |
| 01SD157 | 89.2 | 5.13 | | | [69] |
| 02SD294 | 89.9 | 5.13 | | | |
| DX-4 c1a | 88.8 | 3.72 | 3625 | 1.42 | [63] |
| DX-4 c2a | 90.0 | 3.56 | 2556 | 1.44 | |
| DX-4 r1a | 87.7 | 3.68 | 3349 | 1.12 | |
| DX-4r2a | 87.1 | 3.66 | 2375 | 1.35 | |
| DX11c1a | 91.8 | 8.71 | 2233 | 4.51 | |
| DX11c2a | 90.7 | 6.26 | 3039 | 4.87 | |
| 03LG-01 | 92.0 | 8.65 | 950 | 1.55 | [70] |
| 03LG-03 | 90.0 | 3.50 | 6171 | 0.34 | |
| 03LG-08 | 89.0 | 3.49 | 4950 | 0.36 | |
| 03LG-10 | 89.0 | 3.62 | 5486 | 0.30 | |
| 03LG-11 | 90.0 | 3.60 | 4800 | 0.82 | |
| LG-10 | 86.0 | 3.19 | 4140 | 0.63 | |
| 03LG-12 | 90.0 | 3.50 | 5571 | 0.31 | |
| 03LG-16 | 89.0 | 3.57 | | | |
| 04LG-9 | 90.0 | 3.51 | | | |
| 04LG-2 | 90.0 | 4.96 | | | |
| 04LG-8 | 90.0 | 3.73 | | | |
| 04LG-10 | 90.0 | 3.62 | | | |
| 04LG-11 | 91.0 | 4.53 | | | |
| 04LG-12 | 90.0 | 3.66 | | | |
| 04LG-01 | 90.0 | 3.36 | | | |
| 04LG-14 | 89.0 | 3.58 | | | |

**Table S6.** Parameters for calculating $CO_2$ contents in melt inclusions of studied lamprophyres.

| Sample No. | Fermi band band $\upsilon^+$ ($cm^{-1}$) | Fermi band band $\upsilon^-$ ($cm^{-1}$) | $\Delta$ ($cm^{-1}$) | $d$ ($g/cm^3$) | Length (μm) | Width (μm) | Inclusion volume ($cm^3$) |
|---|---|---|---|---|---|---|---|
| MI-01 | 1283.70 | 1387.30 | 103.60 | 0.47 | 29.01 | 14.08 | 5.75E-09 |
| MI-02 | 1284.14 | 1387.74 | 103.60 | 0.47 | 38.13 | 16.82 | 1.08E-08 |
| MI-03 | 1284.14 | 1387.30 | 103.16 | 0.28 | 7.72 | 7.28 | 4.09E-10 |
| MI-04 | 1283.26 | 1387.30 | 104.04 | 0.67 | 6.80 | 5.49 | 2.05E-10 |
| MI-05 | 1283.26 | 1387.30 | 104.04 | 0.67 | 4.45 | 4.07 | 7.37E-11 |
| MI-06 | 1283.70 | 1387.30 | 103.60 | 0.47 | 10.77 | 9.09 | 8.90E-10 |
| MI-07 | 1284.14 | 1387.74 | 103.60 | 0.47 | 13.57 | 11.41 | 1.77E-09 |
| MI-08 | 1284.14 | 1387.74 | 103.60 | 0.47 | 6.98 | 5.46 | 2.08E-10 |
| MI-09 | 1283.26 | 1387.30 | 104.04 | 0.67 | 10.49 | 8.05 | 6.80E-10 |
| MI-10 | 1282.82 | 1387.30 | 104.48 | 0.86 | 5.12 | 4.32 | 9.56E-11 |
| MI-11 | 1283.70 | 1387.74 | 104.04 | 0.67 | 9.86 | 3.11 | 9.54E-11 |
| MI-12 | 1283.70 | 1387.30 | 103.60 | 0.47 | 7.69 | 4.4 | 1.49E-10 |
| MI-13 | 1283.26 | 1386.43 | 103.17 | 0.28 | 3.43 | 2.35 | 1.89E-11 |
| MI-14 | 1282.37 | 1386.87 | 104.50 | 0.87 | 7.63 | 5.13 | 2.01E-10 |
| MI-15 | 1284.14 | 1387.74 | 103.60 | 0.47 | 8.17 | 3.2 | 8.37E-11 |
| MI-16 | 1283.26 | 1387.30 | 104.04 | 0.67 | 11.23 | 5.87 | 3.87E-10 |
| MI-17 | 1283.26 | 1387.30 | 104.04 | 0.67 | 3.46 | 3.16 | 3.46E-11 |
| MI-18 | 1284.59 | 1387.30 | 102.71 | 0.11 | 5.47 | 2.24 | 2.74E-11 |
| MI-19 | 1283.70 | 1387.30 | 103.60 | 0.47 | 14.79 | 5.12 | 3.88E-10 |
| MI-20 | 1285.03 | 1388.17 | 103.14 | 0.27 | 9.03 | 7.35 | 4.88E-10 |
| MI-21 | 1284.14 | 1387.30 | 103.16 | 0.28 | 5.81 | 4.13 | 9.91E-11 |
| MI-22 | 1285.47 | 1388.17 | 102.70 | 0.10 | 13.86 | 10.65 | 1.57E-09 |
| MI-23 | 1283.70 | 1387.30 | 103.60 | 0.47 | 8.82 | 4.80 | 2.03E-10 |
| MI-24 | 1284.14 | 1387.30 | 103.16 | 0.28 | 15.78 | 8.88 | 1.24E-09 |
| MI-25 | 1283.26 | 1387.30 | 104.04 | 0.67 | 10.91 | 5.96 | 3.88E-10 |
| MI-26 | 1283.26 | 1387.30 | 104.04 | 0.67 | 12.55 | 12.07 | 1.83E-09 |
| MI-27 | 1283.70 | 1387.30 | 103.60 | 0.47 | 12.48 | 11.25 | 1.58E-09 |
| MI-28 | 1282.82 | 1386.87 | 104.05 | 0.67 | 7.86 | 7.16 | 4.03E-10 |
| MI-29 | 1282.37 | 1386.87 | 104.50 | 0.87 | 5.95 | 4.25 | 1.07E-10 |

Continued

| Diameter (μm) | Bubbe volume ($cm^3$) | Bubble (vol.%) | $M_{vb}^{CO_2}$ (g) | Glass volume ($cm^3$) | $M_{gl}$ (g) | [$CO_2$] in bubble (ppm) | [$CO_2$] in MI (ppm) | [$CO_2$] in MI (%) |
|---|---|---|---|---|---|---|---|---|
| 3.17 | 1.67E-11 | 0.29 | 7.82E-12 | 5.73E-09 | 1.58E-08 | 496 | 647 | 0.0647 |
| 3.39 | 2.04E-11 | 0.19 | 9.57E-12 | 1.08E-08 | 2.96E-08 | 323 | 474 | 0.0474 |
| 2.37 | 6.97E-12 | 1.70 | 1.93E-12 | 4.02E-10 | 1.11E-09 | 1749 | 1900 | 0.1900 |
| 3.63 | 2.50E-11 | 12.22 | 1.67E-11 | 1.80E-10 | 4.95E-10 | 33804 | 33955 | 3.3955 |
| 1.24 | 9.98E-13 | 1.35 | 6.67E-13 | 7.27E-11 | 2.00E-10 | 3334 | 3485 | 0.3485 |
| 5.86 | 1.05E-10 | 11.84 | 4.94E-11 | 7.85E-10 | 2.16E-09 | 22910 | 23061 | 2.3061 |
| 5.34 | 7.97E-11 | 4.51 | 3.74E-11 | 1.69E-09 | 4.64E-09 | 8063 | 8214 | 0.8214 |
| 2.43 | 7.51E-12 | 3.61 | 3.52E-12 | 2.01E-10 | 5.52E-10 | 6390 | 6541 | 0.6541 |
| 5.80 | 1.02E-10 | 15.03 | 6.82E-11 | 5.78E-10 | 1.59E-09 | 42948 | 43099 | 4.3099 |
| 2.76 | 1.10E-11 | 11.52 | 9.43E-12 | 8.45E-11 | 2.32E-10 | 40579 | 40730 | 4.0730 |
| 2.45 | 7.70E-12 | 8.07 | 5.14E-12 | 8.77E-11 | 2.41E-10 | 21328 | 21479 | 2.1479 |
| 2.80 | 1.15E-11 | 7.72 | 5.39E-12 | 1.37E-10 | 3.78E-10 | 14272 | 14423 | 1.4423 |
| 1.75 | 2.81E-12 | 14.81 | 7.91E-13 | 1.61E-11 | 4.44E-11 | 17818 | 17969 | 1.7969 |
| 2.44 | 7.61E-12 | 3.79 | 6.58E-12 | 1.93E-10 | 5.31E-10 | 12386 | 12537 | 1.2537 |
| 2.92 | 1.30E-11 | 15.58 | 6.12E-12 | 7.06E-11 | 1.94E-10 | 31488 | 31639 | 3.1639 |
| 3.59 | 2.42E-11 | 6.26 | 1.62E-11 | 3.63E-10 | 9.97E-10 | 16218 | 16369 | 1.6369 |
| 2.21 | 5.65E-12 | 16.36 | 3.77E-12 | 2.89E-11 | 7.95E-11 | 47490 | 47641 | 4.7641 |
| 1.05 | 6.06E-13 | 2.21 | 6.45E-14 | 2.68E-11 | 7.38E-11 | 873 | 1024 | 0.1024 |
| 3.55 | 2.34E-11 | 6.04 | 1.10E-11 | 3.64E-10 | 1.00E-09 | 10970 | 11121 | 1.1121 |
| 3.49 | 2.23E-11 | 4.56 | 5.99E-12 | 4.66E-10 | 1.28E-09 | 4682 | 4833 | 0.4833 |
| 1.40 | 1.44E-12 | 1.45 | 3.99E-13 | 9.77E-11 | 2.69E-10 | 1485 | 1636 | 0.1636 |
| 5.23 | 7.49E-11 | 4.76 | 7.71E-12 | 1.50E-09 | 4.12E-09 | 1873 | 2024 | 0.2024 |
| 4.11 | 3.64E-11 | 17.89 | 1.71E-11 | 1.67E-10 | 4.59E-10 | 37164 | 37315 | 3.7315 |
| 7.31 | 2.05E-10 | 16.44 | 5.68E-11 | 1.04E-09 | 2.86E-09 | 19856 | 20007 | 2.0007 |
| 4.28 | 4.11E-11 | 10.59 | 2.74E-11 | 3.46E-10 | 9.53E-10 | 28770 | 28921 | 2.8921 |
| 6.16 | 1.22E-10 | 6.69 | 8.17E-11 | 1.71E-09 | 4.69E-09 | 17421 | 17572 | 1.7572 |
| 6.06 | 1.17E-10 | 7.38 | 5.47E-11 | 1.46E-09 | 4.02E-09 | 13587 | 13738 | 1.3738 |
| 2.94 | 1.33E-11 | 3.30 | 8.94E-12 | 3.90E-10 | 1.07E-09 | 8348 | 8499 | 0.8499 |
| 2.04 | 4.45E-12 | 4.14 | 3.85E-12 | 1.03E-10 | 2.83E-10 | 13573 | 13724 | 1.3724 |

## References

1. Liu D Y, Nutman A P, Compston W, *et al.* Remnants of ⩾ 3800 Ma crust in the Chinese part of the Sino-Korean craton. *Geology* 1992; **20**: 339-42.
2. Zhao G C, Wilde S A, Cawood P A, *et al.* Petrology and P–T path of the Fuping mafic granulites: implications for tectonic evolution of the central zone of the North China craton. *J Metamorph Geol* 2000; **18**: 375-91.
3. Zhao G C, Wilde S A, Cawood P A, *et al.* Archean blocks and their boundaries in the North China Craton: lithological, geochemical, structural and P–T path constraints and tectonic evolution. *Precambrian Res* 2001; **107**: 45-73.
4. Menzies M, Xu Y, Zhang H, *et al.* Integration of geology, geophysics and geochemistry: A key to understanding the North China Craton. *Lithos* 2007; **96**: 1-21.
5. Menzies M A, Fan W, Zhang M. Palaeozoic and Cenozoic lithoprobes and the loss of > 120 km of Archaean lithosphere, Sino-Korean craton, China. *Geol Soc Spec Publ* 1993; **76**: 71-81.
6. Griffin W L, Zhan A, O'Reilly S Y, *et al.* PhanerozoiEcv olutiono f the Lithosphere Beneath the S ino-Korean Craton. *Magmatic Processes and Plate Tectonic* 1998; **27**: 107-26.
7. Gao S, Rudnick R L, Carlson R W, *et al.* Re–Os evidence for replacement of ancient mantle lithosphere beneath the North China craton. *Earth Planet Sc Lett* 2002; **198**: 307-22.
8. Fan W, Menzies M. Destruction of aged lower lithosphere and accretion of asthenosphere mantle beneath eastern China. *Phys Chem Earth* 1992: 171-80.
9. Liu J, Cai R, Pearson D G, *et al.* Thinning and destruction of the lithospheric mantle root beneath the North China Craton: A review. *Earth-Science Reviews* 2019; **196**.
10. Xu L-J, Liu S-A, Li S. Zinc isotopic behavior of mafic rocks during continental deep subduction. *Geoscience Frontiers* 2021; **12**.
11. Ke S, Teng F-Z, Li S-G, *et al.* Mg, Sr, and O isotope geochemistry of syenites from northwest Xinjiang, China: Tracing carbonate recycling during Tethyan oceanic subduction. *Chem Geol* 2016; **437**: 109-19.
12. Galy A, Yoffe O, Janney P E, *et al.* Magnesium isotope heterogeneity of the isotopic standard SRM980 and new reference materials for magnesium-isotope-ratio measurements. *J Anal Atom Spectrom* 2003; **18**.
13. Teng F-Z, Li W-Y, Ke S, *et al.* Magnesium Isotopic Compositions of International Geological Reference Materials. *Geostand Geoanal Res* 2015; **39**: 329-39.
14. Yang J-H, Chung S-L, Zhai M-G, *et al.* Geochemical and Sr–Nd–Pb isotopic compositions of mafic dikes from the Jiaodong Peninsula, China: evidence for vein-plus-peridotite melting in the lithospheric mantle. *Lithos* 2004; **73**: 145-60.
15. Ma L, Jiang S-Y, Hofmann A W, *et al.* Lithospheric and asthenospheric sources of lamprophyres in the Jiaodong Peninsula: A consequence of rapid lithospheric thinning beneath the North China Craton? *Geochim Cosmochim Acta* 2014; **124**: 250-71.
16. Ma L, Jiang S-Y, Hofmann A W, *et al.* Rapid lithospheric thinning of the North China Craton: New evidence from cretaceous mafic dikes in the Jiaodong Peninsula. *Chem Geol* 2016; **432**: 1-15.
17. Li X-Y, Li S-Z, Suo Y-H, *et al.* Early Cretaceous diabases, lamprophyres and andesites-dacites in western Shandong, North China Craton: Implications for local delamination and Paleo-Pacific slab rollback. *J Asian Earth Sci* 2018; **160**: 426-44.
18. Yang H-T, Yang D-B, Mu M-S, *et al.* Sr–Nd–Hf isotopic compositions of lamprophyres in western Shandong, China: Implications for the nature of the early cretaceous lithospheric mantle beneath the eastern North China Craton. *Lithos* 2019; **336-337**: 1-13.

19. Wang X, Wang Z, Cheng H, *et al.* Early cretaceous lamprophyre dyke swarms in Jiaodong Peninsula, eastern North China Craton, and implications for mantle metasomatism related to subduction. *Lithos* 2020; **368-369**.
20. Rudnick R L, Founatin D M. Nature and composition of the continental crust: a lower crustal perspective. *Reviews of Geophysics* 1995; **33**: 267-309.
21. Ma L, Jiang S-Y, Dai B-Z, *et al.* Multiple sources for the origin of Late Jurassic Linglong adakitic granite in the Shandong Peninsula, eastern China: Zircon U–Pb geochronological, geochemical and Sr–Nd–Hf isotopic evidence. *Lithos* 2013; **162-163**: 251-63.
22. Teng F-Z. Magnesium Isotope Geochemistry. *Rev Mineral Geochem* 2017; **82**: 219-87.
23. Dasgupta R, Hirschmann M M, Smith N D. Partial Melting Experiments of Peridotite + CO2 at 3 GPa and Genesis of Alkalic Ocean Island Basalts. *Journal of Petrology* 2007; **48**: 2093-124.
24. Su B-X, Hu Y, Teng F-Z, *et al.* Chromite-induced magnesium isotope fractionation during mafic magma differentiation. *Science Bulletin* 2017; **62**: 1538-46.
25. Steele-MacInnis M, Esposito R, Moore L R, *et al.* Heterogeneously entrapped, vapor-rich melt inclusions record pre-eruptive magmatic volatile contents. *Contrib to Mineral Petrol* 2017; **172**.
26. Capriolo M, Marzoli A, Aradi L E, *et al.* Deep CO2 in the end-Triassic Central Atlantic Magmatic Province. *Nat Commun* 2020; **11**: 1670.
27. Hartley M E, Maclennan J, Edmonds M, *et al.* Reconstructing the deep CO2 degassing behaviour of large basaltic fissure eruptions. *Earth Planet Sc Lett* 2014; **393**: 120-31.
28. Frezzotti M L, Tecce F, Casagli A. Raman spectroscopy for fluid inclusion analysis. *J Geochem Explor* 2012; **112**: 1-20.
29. Kawakami Y, Yamamoto J, Kagi H. Micro-Raman Densimeter for $CO_2$ inclusions in mantle-derived minerals. *Appl Spectrosc* 2003; **57**: 1333-9.
30. Rosso K M, Bodnar R J. Microthermometric and Raman spectroscopic detection limits of $CO_2$ in fluid inclusions and the Raman spectroscopic characterization of $CO_2$. *Geochimica et Cosmochimica Acta* 1995; **59**: 3961–75.
31. Song Y, Chou I M, Hu W, *et al.* $CO_2$ density-Raman shift relation derived from synthetic inclusions in fused silica capillaries and its application. *Acta Geol Sin* 2009; **83**: 932–8.
32. Wang X, Chou I M, Hu W, *et al.* Raman spectroscopic measurements of CO2 density: Experimental calibration with high-pressure optical cell (HPOC) and fused silica capillary capsule (FSCC) with application to fluid inclusion observations. *Geochim Cosmochim Acta* 2011; **75**: 4080-93.
33. Fall A, Tattitch B, Bodnar R J. Combined microthermometric and Raman spectroscopic technique to determine the salinity of H2O–CO2–NaCl fluid inclusions based on clathrate melting. *Geochim Cosmochim Acta* 2011; **75**: 951-64.
34. Deng J, Liu X, Wang Q, *et al.* Isotopic characterization and petrogenetic modeling of Early Cretaceous mafic diking—Lithospheric extension in the North China craton, eastern Asia. *GSA Bulletin* 2017; **129**: 1379-407.
35. Ma L, Jiang S-Y, Hou M-L, *et al.* Geochemistry of Early Cretaceous calc-alkaline lamprophyres in the Jiaodong Peninsula: Implication for lithospheric evolution of the eastern North China Craton. *Gondwana Res* 2014; **25**: 859-72.
36. Li C, Yan J. Geochemical, mineralogy, and Sr–Nd–Pb isotopic compositions of the gold-related lamprophyre in the Bengbu–Wuhe Area, southeastern North China Craton: Implications for gold mineralization. *Ore Geology Reviews* 2021; **132**.

37. Guo F, Fan W, Wang Y, *et al.* Origin of early Cretaceous calc-alkaline lamprophyres from the Sulu orogen in eastern China: implications for enrichment processes beneath continental collisional belt. *Lithos* 2004; **78**: 291-305.
38. Chen B, Zhai M. Geochemistry of late Mesozoic lamprophyre dykes from the Taihang Mountains, north China, and implications for the sub-continental lithospheric mantle. *Geological Magazine* 2003; **140**: 87-93.
39. Wu F, Xiao Y, Xu L, *et al.* Geochronology and geochemistry of felsic xenoliths in lamprophyre dikes from the southeastern margin of the North China Craton: implications for the interleaving of the Dabie–Sulu orogenic crust. *Int Geol Rev* 2015; **57**: 1305-25.
40. Li C, Li L, Li S-R, *et al.* Mesozoic mafic dykes in the North China Craton: magmatic evolution and implications for gold mineralization. *Int Geol Rev* 2021: 1-21.
41. Hong L, Xu Y-G, Zhang L, *et al.* Recycled carbonate-induced oxidization of the convective mantle beneath Jiaodong, Eastern China. *Lithos* 2020; **366-367**.
42. Gao S, Rudnick R L, Xu W-L, *et al.* Recycling deep cratonic lithosphere and generation of intraplate magmatism in the North China Craton. *Earth Planet Sc Lett* 2008; **270**: 41-53.
43. Li S-G, Yang W, Ke S, *et al.* Deep carbon cycles constrained by a large-scale mantle Mg isotope anomaly in eastern China. *Natl Sci Rev* 2017; **4**: 111-20.
44. Yang W, Li S. Geochronology and geochemistry of the Mesozoic volcanic rocks in Western Liaoning: Implications for lithospheric thinning of the North China Craton. *Lithos* 2008; **102**: 88-117.
45. Li W, Li X, Lu F, *et al.* Geological characteristics and its setting for volcanic rocks of early Cretaceous Yixian Formation in western Liaoning province, eastern China. *Acta Petrol Sin* 2002; **2**: 193-204.
46. Feng Y, Yang J, Sun J, *et al.* Material records for Mesozoic destruction of the North China Craton by subduction of the Paleo-Pacific slab. *Sci China Earth Sci* 2020; **63**: 690-700.
47. Wang F. Geochemistry and geochronology of Dabeigou volcanic rocks in Chengde Basin, Hebei Province. *Master Thesis. Type*. North west University, 2007.
48. Liu J. The geochemistry of late Mesozoic volcanic rocks from the North CHina Craton. *Master Thesis. Type*. Lanzhou University 2015.
49. Fan W M, Guo F, Wang Y J, *et al.* Post-orogenic bimodal volcanism along the Sulu Orogenic belt in eastern China. *Phys Chem Earth (A)* 2001; **26**: 733-46.
50. Kuang Y, Pang C, Luo Z, *et al.* $^{40}$Ar-$^{39}$Ar geochronology and geochemistry of mafic rocks from Qingshan Group, Jiaodong area: Implications for the destruction of the North China Craton. *Acta Petrol Sin* 2012; **4**: 1073-91.
51. Lin B. Geochemistry and petrogenesis of Mesozoic volcanic rocks in the geological corridor of western Liaoning. *Doctor Thesis. Type*. Jilin University, 2017.
52. Workmana R K, Hart S R. Major and trace element composition of the depleted MORB mantle (DMM). *Earth Planet Sc Lett* 2005; **231**: 53-72.
53. Teng F-Z, Li W-Y, Ke S, *et al.* Magnesium isotopic composition of the Earth and chondrites. *Geochim Cosmochim Acta* 2010; **74**: 4150-66.
54. Huang J, Xiao Y. Mg-Sr isotopes of low-δ26Mg basalts tracing recycled carbonate species: Implication for the initial melting depth of the carbonated mantle in Eastern China. *Int Geol Rev* 2016; **58**: 1350-62.
55. Plank T. The Chemical Composition of Subducting Sediments. *Treatise on Geochemistry*2014, 607-29.
56. Wang S-J, Teng F-Z, Rudnick R L, *et al.* The behavior of magnesium isotopes in low-grade metamorphosed mudrocks. *Geochim Cosmochim Acta* 2015; **165**: 435-48.

57. Zheng J, Griffin W L, O'Reilly S Y, *et al.* Mineral Chemistry of Peridotites from Paleozoic, Mesozoic and Cenozoic Lithosphere: Constraints on Mantle Evolution beneath Eastern China. *Journal of Petrology* 2006; **47**: 2233-56.
58. Zheng J, Sun M, Zhou M-F, *et al.* Trace elemental and PGE geochemical constraints of Mesozoic and Cenozoic peridotitic xenoliths on lithospheric evolution of the North China Craton. *Geochim Cosmochim Acta* 2005; **69**: 3401-18.
59. Xu W, Yang D, Gao S, *et al.* Geochemistry of peridotite xenoliths in Early Cretaceous high-Mg# diorites from the Central Orogenic Block of the North China Craton: The nature of Mesozoic lithospheric mantle and constraints on lithospheric thinning. *Chem Geol* 2010; **270**: 257-73.
60. Xu W, Hergt J M, Gao S, *et al.* Interaction of adakitic melt-peridotite: Implications for the high-Mg# signature of Mesozoic adakitic rocks in the eastern North China Craton. *Earth Planet Sc Lett* 2008; **265**: 123-37.
61. Zhou Q, Xu W, Yang D, *et al.* Modification of the lithospheric mantle by melt derived from recycled continental crust evidenced by wehrlite xenoliths in Early Cretaceous high-Mg diorites from western Shandong, China. *Sci China Earth Sci* 2012; **55**: 1972-86.
62. Zhou Q-J. Petrogenesis of wehrlite and pyroxenite xenolites in early Cretaceous igneous rocks from western Shandong, China. *Thesis. Type*2014.
63. Xu W-L, Zhou Q-J, Pei F-P, *et al.* Destruction of the North China Craton: Delamination or thermal/chemical erosion? Mineral chemistry and oxygen isotope insights from websterite xenoliths. *Gondwana Res* 2013; **23**: 119-29.
64. Zou D, Zhang H, Zhang X, *et al.* Refertilization of lithospheric mantle beneath the North China Craton in Mesozoic: Evidence from in situ Sr isotopes of Fuxin peridotite. *Lithos* 2020; **364-365**.
65. Zheng J P, Griffin W L, O'Reilly S Y, *et al.* Mechanism and timing of lithospheric modification and replacement beneath the eastern North China Craton: Peridotitic xenoliths from the 100 Ma Fuxin basalts and a regional synthesis. *Geochim Cosmochim Acta* 2007; **71**: 5203-25.
66. Lu S-m, Pei F-P, Zhou Q-J, *et al.* Origin of late Mesozoic alkaline basalts and nature of lithospheric mantle in Liaoyuan area,Jilin Province. *Earth Sci J China Univ Geosci* 2012; **37**: 476-88.
67. Zhang J, Zhang H, Kita N, *et al.* Secular evolution of the lithospheric mantle beneath the eastern North China craton: evidence from peridotitic xenoliths from Late Cretaceous mafic rocks in the Jiaodong region, east-central China. *Int Geol Rev* 2011; **53**: 182-211.
68. Zhao X, Wang H, Li Z, *et al.* Multi-stage metasomatism of lithospheric mantle by asthenosphere-derived melts: evidence from mantle xenoliths in daxizhuang at the eastern North China craton. *Miner Petrol* 2020; **114**: 141-59.
69. Yan J, Cheng J, Xie Z, *et al.* Mantlo-derived xenoliths from the Late Cretaceous basalts in eastern Ludong: New evidence for time constraints on lithospheric thinning in eastern China. *Sci Bull* 2003; **48**: 1570-4.
70. Ying J, Zhang H, Kita N, *et al.* Nature and evolution of Late Cretaceous lithospheric mantle beneath the eastern North China Craton: Constraints from petrology and geochemistry of peridotitic xenoliths from Jünan, Shandong Province, China. *Earth Planet Sc Lett* 2006; **244**: 622-38.
